# Metabolomic profiling of backfat in Ningxiang pigs reveals lipid dynamics and carcass trait associations during the fattening stage

**DOI:** 10.1371/journal.pone.0353743

**Published:** 2026-07-22

**Authors:** Yu Chen, Lihua Cao, Qingming Cui, Yuan Deng, Ji Zhu, Yingying Liu, Huali Li, Huibo Ren, Xionggui Hu, Xiaogang Zhao, Xinglong He, Huiming Wang, Wenmei Wang, Yinglin Peng, Chen Chen

**Affiliations:** 1 Institute of Animal and Veterinary Medicine, Hunan, China; 2 Yuelushan Laboratory, Changsha, Hunan, China; 3 Animal Disease Prevention and Control Center, Changsha, Hunan, China; 4 Hunan Liushahe Ecological Animal Husbandry Co., Ltd, Changsha, Hunan, China; University of Agriculture Faisalabad, PAKISTAN

## Abstract

Understanding the metabolic profile of backfat is essential for optimizing breeding strategies and improving pork production. While the early stages of adipose tissue development have been partially characterized, metabolic alterations during the fattening phase (180–360 days)— a critical period for physiological maturation and carcass trait formation—remain insufficiently understood. In this study, we characterized the metabolomic profiles of backfat from Ningxiang pigs across four developmental stages (180d, 240d, 300d, 360d) and evaluated their relationships with carcass traits. A total of 154 metabolites exhibited significant temporal variation (q < 0.05), including functional lipids such as methanandamide phosphate and oleoylethanolamide, which are associated with appetite regulation and lipid metabolism. The progressive accumulation of bioactive exogenous metabolites like α-tocotrienol and cephaeline, indicated potential stage-dependent immunometabolic adaptations. In contrast, several synthetic xenobiotics, potentially derived from feed additives or environmental exposure, accumulated in backfat tissue and may represent potential risks to animal health and pork safety. Co-expression network analysis identified a metabolite module strongly associated (|R| > 0.7, q < 0.05) with key carcass traits, within which psychotrine—an understudied plant-derived alkaloid not previously associated with animal growth— was identified as a hub metabolite. This study establishes a comprehensive metabolic characterization of backfat during the fattening phase in an indigenous pig breed. Collectively, these findings provide novel candidate biomarkers for carcass trait prediction, highlight the potential impact of synthetic compound accumulation, and offer valuable insights for precision breeding, nutritional management, and meat safety assessment in swine production.

## Introduction

Ningxiang pigs, a well-recognized indigenous breed in China, are characterized by superior fat deposition capacity, distinctive fatty acid composition, and unique adipose tissue metabolic characteristics [1, 2]. Their high intramuscular fat and backfat content contribute substantially to desirable meat quality attributes, particularly flavor, juiciness, and texture, making this breed an important genetic resource for premium pork production and breeding programs [-1,3–5]. Therefore, a comprehensive understanding of the adipose tissue metabolome in Ningxiang pigs is critical for the conservation of their unique genetic characteristics and for facilitating future genetic improvement strategies.

Backfat thickness is an important indicator in pig breeding and pork production and is widely applied in the evaluation of carcass composition, meat quality, and production efficiency [6–8]. Adipose tissue functions not only as a storage site for fatty acids but also as an active metabolic and endocrine organ involved in the regulation of endocrine signaling [9, 10], energy homeostasis [11], and immune responses [12–14]. In addition, adipose tissue serves as a reservoir for various lipophilic compounds [15] and bioactive signaling lipids that participate in metabolic regulation [16]. Recent multi-omics studies have provided valuable insights into the complex genetic, proteomic and metabolic interactions governing adipose tissue composition and its impact on meat quality [1,2,4,17–19].

Ningxiang pigs, similar to many Chinese indigenous pig breeds, reach sexual maturity at a relatively early age (approximately 120 d), whereas complete physiological and body maturation occurs later (around 300-360d). Although the metabolomic characteristics of backfat during the growing stage have previously been investigated in Ningxiang pigs [17], the metabolic profile during the fattening stage remains poorly understood. The fattening stage (180–360d) is particularly important because it is closely associated with economically significant traits, including fat deposition and meat quality. Therefore, focusing on this developmental window enables a systematic characterization of temporal metabolic alterations in backfat tissue and provides a scientific foundation for optimizing nutritional management and production strategies in Ningxiang pigs.

This study characterized the metabolomic profile of backfat in Ningxiang pigs at four later developmental stages (180d, 240d, 300d and 360d) and conducted pairwise comparison among stages to identify candidate metabolites associated with lipid metabolism and adipose tissue development. In addition, a module–trait correlation network was constructed based on metabolomic data to evaluate the relationships between metabolite modules and key carcass traits. We hypothesize that stage-dependent alterations in the backfat metabolome of Ningxiang pigs reflect coordinated regulation of energy metabolism and that specific metabolites are associated with economically important carcass traits and breeding performance.

## Materials and methods

### Animal ethics

Throughout the experimental period, Ningxiang pigs were provided with *ad libitum* access to clean water and a nutritionally balanced diet. The ingredient composition and proximate nutritional composition of the diet were provided in S1 Table. Animals were monitored daily for signs of illness or distress, and any sick or injured animals received prompt veterinary care in accordance with institutional guidelines. At the end of the experiment, pigs were humanely euthanized using electrical stunning followed by slaughter. All animal procedures in this study were conducted in strict accordance with the guidelines of the Animal Care and Use Committee of the Hunan Institute of Animal Husbandry and Veterinary Medicine (Approval No. 20211015).

### Animals and tissue collection

Twenty-four Ningxiang pigs at four developmental stages (180, 240, 300, and 360 days) were provided by Hunan Liushahe Ecological Animal Husbandry Co., Ltd, and all originated from the same closed breeding population to minimize genetic variation among individuals. Backfat tissue was collected within 30 minutes post-mortem from the thickest region over the shoulder, snap-frozen in liquid nitrogen, and stored at −80°C for subsequent metabolomic analysis.

### Metabolite extraction

Fifty milligrams of backfat tissue from each animal were used for metabolite extraction. The tissues were ground with a mortar and pestle in liquid nitrogen and then transferred to a 1.8 mL Eppendorf tube containing 1 mL of pre-cooled extraction solution (50% methanol:acetonitrile:water, 2:2:1). Samples were thoroughly homogenized and incubated at room temperature for 10 minutes, and then stored in a −20°C freezer overnight to facilitate proper quenching, extraction, and protein precipitation. On the following day, the solutions were centrifuged at 14,000 × g for 15 minutes at 4°C, and the supernatant was transferred to a new tube for LC-MS analysis. In addition, pooled quality control samples were prepared by mixing 10 μL of extract from each extract sample.

### LC-MS/MS profiling

LC-MS analysis was conducted using an UltiMate 3000 HPLC (Thermo Scientific, USA) equipped with an ACQUITY UPLC BEH C18 column (1.8μm, 2.1 mm*100 mm, Waters, USA) maintained at 35°C. The injection volume for each sample was 4 μL. The flow rate was set at 0.4 ml/min and the mobile phase consisted of solvent A (water, 0.1% formic acid) and solvent B (acetonitrile, 0.1% formic acid). Gradient elution conditions were set as follows: 0 ～ 0.5 min, 5% solvent B; 0.5 ～ 7 min, 5% to 100% solvent B; 7 ~ 8 min, 100% solvent B; 8 ～ 8.1 min, 100% to 5% solvent B; 8.1 ～ 10 min, 5% solvent B.

Metabolites eluted from the column were detected with a high-resolution tandem mass spectrometer Q-Exactive (Thermo Scientific, US), operating in both positive and negative ion modes using electrospray ionization. Precursor spectra (m/z: 70–1050) were acquired at a resolution of 70,000 with an AGC target of 3e6 and a maximum injection time of 100 ms. Data were acquired in data-dependent acquisition mode with a ‘Top 3’ configuration, selecting the three most intense precursor ions for fragmentation per cycle. MS/MS spectra were acquired at a resolution of 17,500 with an AGC target of 1e5 and a maximum injection time of 80 ms. A pooled quality control (QC) sample was injected after every 10 samples to monitor instrument stability and reproducibility during LC-MS analysis. QC assessment on raw intensities showed that 3 826 of 3 913 features (97.8%) passed the RSD cutoff of 30%, indicating high reproducibility (S1 Fig). RSD values ranged from 1.0% to 105.1%, with a median of 9.8% and mean of 12.1% (S1 Fig). Principal component analysis (PCA) revealed tight clustering of QC injections (S2 Fig). Total ion current (TIC) was calculated for all QC injections to monitor overall signal stability. TIC values were highly consistent, with a mean of 9.02 × 10¹⁰ and a standard deviation of 1.93 × 10⁹, resulting in a coefficient of variation (CV) of 2.14% (S3 Fig). The TIC plot showed no systematic drift across injections, confirming excellent instrument performance and reproducibility throughout the analytical run. LC − MS raw data were processed with XCMS (version 3.22.0) [20], CAMERA (version 1.54.0) [21] and metaX (version 1.8.0) [22] R package. Each ion was identified based on its retention time (RT) and m/z data. For example, an ion pos-0.865_131.06915 had a RT with 0.865 and m/z with 131.06915. A variable importance in projection (VIP) score of ≥ 1 was used as the cutoff for metabolite identification.

### Identification of metabolites with significant temporal variation

Metabolite annotation was performed with online KEGG [23] and HMDB database [24]. To identify metabolites showing exhibiting significant temporal variation across developmental stages, one-way analysis of variance (ANOVA) was conducted in R [25]. Resulting P-values were adjusted using the Benjamini–Hochberg method, and metabolites adjusted significance levels (q < 0.05) were subsequently subjected to post-hoc pairwise comparisons using Tukey’s honestly significant difference (HSD) test. Boxplots of significantly altered metabolites were generated with ggplot2 (version 3.5.2) [26]. The principal component analysis (PCA) plot and heatmap were drawn with ggplot2 (version 3.5.2) [27] and pheatmap [28] packages. Detailed results of ANOVA and Tukey’s HSD were listed in S1 File.

### Co-abundance network construction with WGCNA

Co-abundance analysis was conducted by weighted gene co-expression network analysis (WGCNA, version 1.73), R package [29]. The R code can be accessed from http://dx.doi.org/10.17504/protocols.io.kqdg3wj51v25/v1. Metabolomic data were normalized (log-transformed) before entering the WGCNA pipeline. A scale-free topology index (R^2^) of 0.9 was used to determine the soft-thresholding power (β). The adjacency matrix was constructed with a power of 9 (β = 9), and the topological overlap matrix was calculated to assess the interconnectedness between metabolites. Modules were merged based on eigengene similarity using a cut height of 0.25 (correlation > 0.75). Details of WGCNA parameters are provided in S2 File. The biological relevance of modules was validated by correlating eigengenes with developmental stages and carcass traits. WGCNA identified 37 different modules, each assigned with a distinct color, with highly correlated metabolites grouped together (S3 File). Hub metabolites were ranked based on module membership values. Our previously published carcass traits data were used to build up module-trait relationships [30] (S4 File). Details of the complete module–trait correlation matrix were provided in S5 File.

To evaluate the robustness of the network analysis, 1,000 permutation tests were performed to verify that significant module–trait associations were unlikely to occur by chance (p < 0.05), thereby supporting reliability of the identified modules. Modules with correlations (|R|) > 0.5 and q < 0.05 were used to construct the module-trait relationship plot using Pearson correlation. Correlations (|R|) > 0.7 were considered significantly associated with carcass traits of interest. A co-abundance network was visualized using Visant [31]. Within each trait-associated module, metabolites were ranked according to their module membership (MM) values. Metabolites with the highest MM values were considered “hub metabolites.” Hub metabolites showing significant correlations with carcass traits (|R| > 0.5, q < 0.05) were further prioritized to ensure network centrality and phenotypic relevance. Additional methodological details regarding metabolite-based co-expression network analysis have been described in previous metabolomics studies [32].

### Statistics

Six biological replicates from each developmental stage were used for metabolomic analysis. The raw intensity values from the metabolomic data were normalized by log transformation. ANOVA was performed to identify metabolites showing significant differences among developmental stages (q < 0.05). P-values were adjusted using the Benjamini-Hochberg correction. Post-hoc testing (Tukey's HSD) was applied to identify significant changes in metabolites between developmental stages. |R| > 0.5 was used as the cutoff, while |R| > 0.7 was considered a strong correlation. All analyses were conducted using R (version 4.4.2)

## Results

### Temporal metabolic shifts revealed by PCA and differential abundance analysis

**Fig 1:**
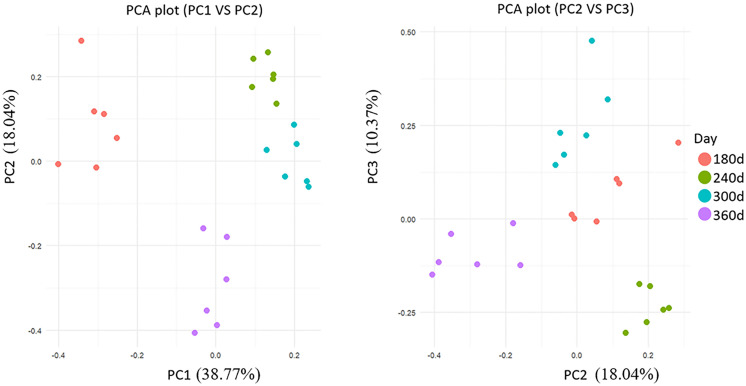
PCA of metabolomic profiles in backfat tissues of Ningxiang pigs at four developmental stages. The PCA revealed clear separation among developmental stages, with samples clustering into three major groups. PC1 distinguished the growing stage (180d) from the fattening stage (240–360d), while PC2 further separated 240d from 360d samples. PC1, PC2, and PC3 together explained 67.18% of the total variance.

Untargeted metabolomic profiling identified a total of 3,913 features in backfat tissues of Ningxiang pigs across four developmental stages (180, 240, 300, and 360 days) (S6 File). PCA revealed distinct separation of samples by age, forming three major groups (Fig 1). Samples collected at 180 d were distinctly separated from those at the later developmental stages (240–360d), contributing predominantly to the variation explained by PC1. PC2 further separated samples from 240 and 360 days, indicating subtler metabolic alterations during later development. Together, PC1, PC2, and PC3 accounted for 67.18% of the total variance, highlighting substantial temporal changes in the backfat metabolome during the fattening phase of Ningxiang pigs.

**Fig 2:**
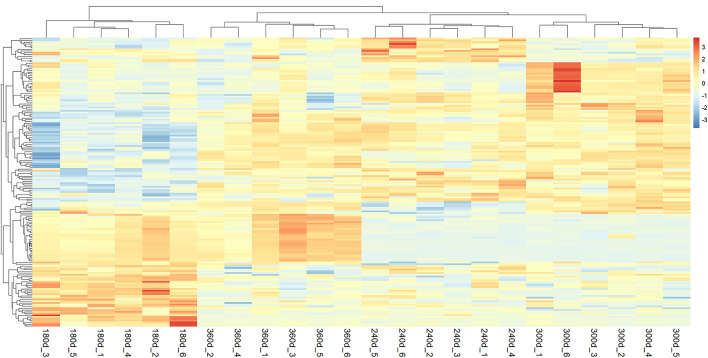
Heatmap of metabolites in the backfat of Ningxiang pigs across 4 developmental stages (180 d, 240d, 300d and 360d). Distinct metabolite clusters (q < 0.05) corresponded to different developmental stages, reflecting dynamic changes in fat metabolism as pigs progress from growth to fattening stage. Rows represented individual metabolites, and columns represented biological replicates at each developmental stage. The heatmap displayed scaled relative abundances (Z-score normalization), with red indicating higher and blue indicating lower metabolite levels. Hierarchical clustering was applied to both metabolites and samples to reveal stage-specific patterns and co-accumulation trends.

Among the 3,913 detected metabolic features, differential abundance analysis was conducted to identify metabolites exhibiting significant temporal variation and to characterize their potential functional relevance. A total of 154 metabolites showed significant differences across the four developmental stages (q < 0.05; Fig 2). Among these, 63 were annotated with KEGG and HMDB databases (MSI 1–2), while the remaining metabolites were putatively annotated (MSI 3–4). All metabolites were classified into three groups based on their properties and biological roles: 1. metabolites involved in lipid metabolism or structural functions; 2. bioactive metabolites, including compounds with known signaling or regulatory roles (e.g., alkaloids, antioxidants); 3. synthetic metabolites, encompassing xenobiotics or artificially derived compounds.

**Fig 3:**
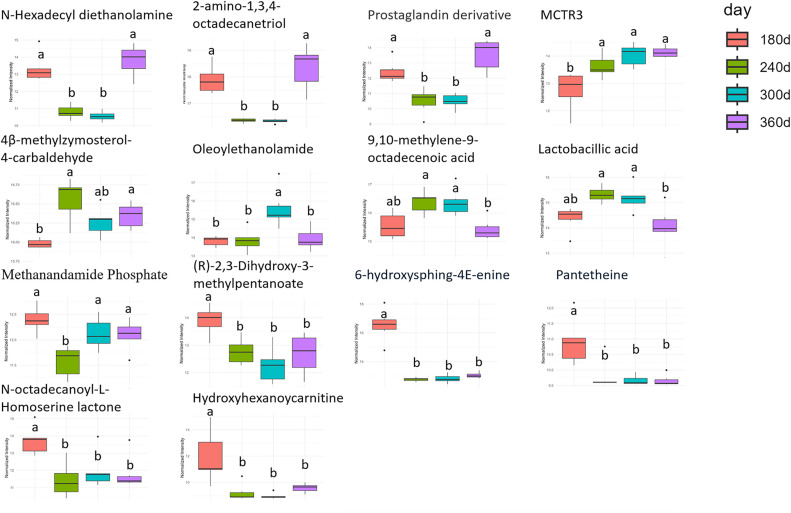
Distribution of differentially abundant lipids in the backfat of Ningxiang pigs across 4 developmental stages. The lipid composition varied significantly (q < 0.05) with age, suggesting stage-specific lipid metabolism changes during pig development. Different letters (a, b, c) above the boxes indicate statistical significance (q < 0.05) (n = 6).

Among identified 63 metabolites, fourteen of them were involved in lipid metabolism and structure: N-Hexadecyl diethanolamine, 2-amino-1,3,4-octadecanetriol and prostaglandin derivative were elevated in backfat at 180d and 360d, MCTR3, 4β-methylzymosterol-4-carbaldehyde, oleoylethanolamide, 9,10,methylene-9-octadecenoic acid, lactobacillic acid showed increased levels at various stages, methanandamide phosphate was decreased at 240d, and (R)-2,3-dihydroxy-3-methylpentanoate, 6-hydroxysphing-4E-enine, pantetheine, N-octadecanoyl-L-homoserine lactone, hydroxyhexanoycarnitine decreased progressively from 180 to 360d (Fig 3). Notably, methanandamide phosphate and oleoylethanolamide, which have opposing effects on appetite and energy homeostasis, showed opposite temporal pattern in backfat. Methanandamide phosphate level was dropped at 240d, while oleoylethanolamide was transiently increased at 300d (Fig 3).

**Fig 4:**
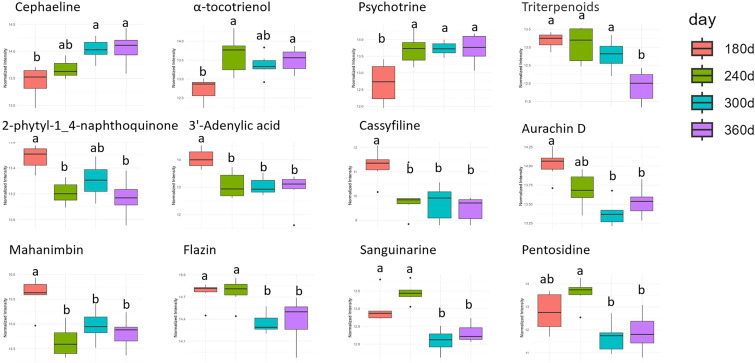
Distribution of differentially abundant bioactive compounds in the backfat of Ningxiang pigs across 4 developmental stages. Changes in bioactive compound levels reflected shifting regulatory and signaling functions in fat tissue over developmental time. Different letters (a, b) above the boxes indicate statistical significance (q < 0.05) (n = 6).

Twelve bioactive metabolites, including signaling alkaloids and antioxidants, were also temporally regulated (Fig 4). For instance, levels of cephaeline, α-tocotrienol (a vitamin E isoform with antioxidant properties) and psychotrine increased from 180 to 360 days (Fig 4). Conversely, triterpenoids, 2-phytyl-1–4-naphthoquinone, 3’-adenylic acid, cassyfiline, aurachin D, mahanimbin, flazin, sanguinarine, and pentosidine were declined over time (Fig 4).

**Fig 5:**
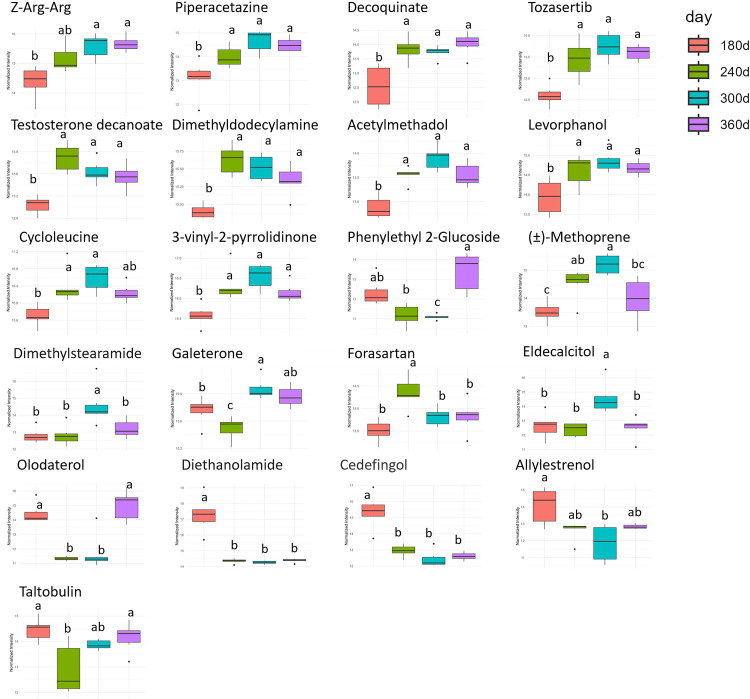
Disstribution of differentially abundant synthetic metabolites in the backfat of Ningxiang pigs across 4 developmental stages. The accumulation of synthetic metabolites over development may pose potential health risks to both the animals and consumers. Different letters (a, b, c) above the boxes indicate statistical significance (q < 0.05) (n = 6).

We also found that twenty-one metabolites were synthetic metabolites, with Z-arg-arg, piperacetazine, decoquinate, tozasertib, testosterone decanoate, dimethyldodecylamine, acetylmethadol, levorphanol, cycloleucine, 3-vinyl-2-pyrrolidinone, phenylethyl 2-glucoside, (±)-methoprene accumulated from 180d to 360d. Dimethylstearamide, galeterone, forasartan, and eldecalcitol were increased at various stage, while olodaterol was decreased at 240d and 300d, diethenolamide, cedefingol, allylestrenol, and taltobulin were gradually decreased in the backfat from 180d to 360d (Fig 5). In addition, we identified two mycotoxins, hydrolyzed fumonisin B1 (HFB1) and paspaline in the backfat. HFB1 was higher in the backfat at 180d and 360d, but paspaline was decreased from 240d (S4 Fig). The function of remaining metabolites was unknown due to lack of information in the database. Detailed information was listed in S7 File.

### Co-abundance network identified metabolite modules associated with carcass traits

**Fig 6:**
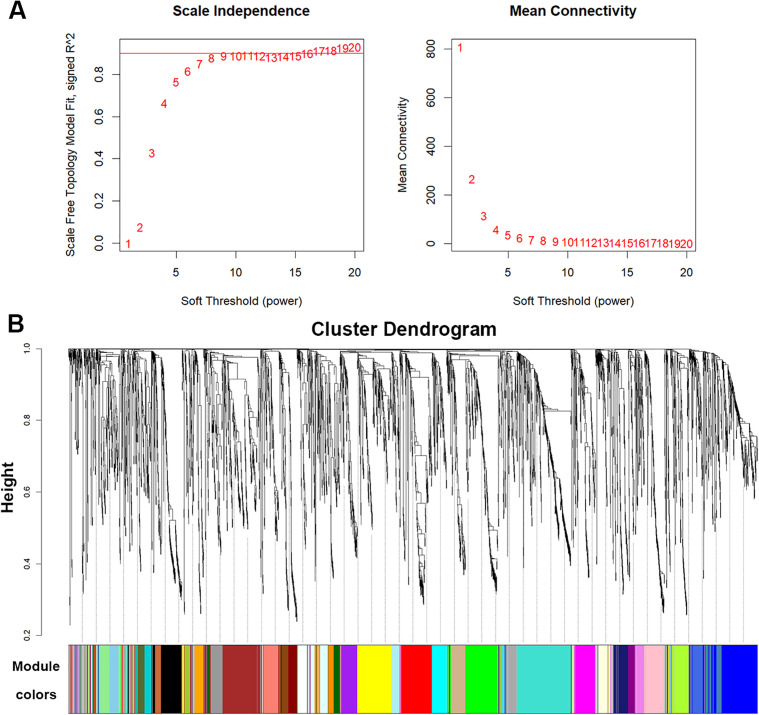
WGCNA network construction with metabolomic data. A cut-off of R^2^ = 0.9 was used to determine the soft-thresholding power (β), and β = 9 was chosen for co-abundance network construction (Fig 6A). Modules were merged based on eigengene similarity using a cut height of 0.25 (correlation > 0.75), while metabolites that did not show apparent patterns were assigned to the grey module. Thirty-seven modules were constructed (Fig 6B).

**Fig 7:**
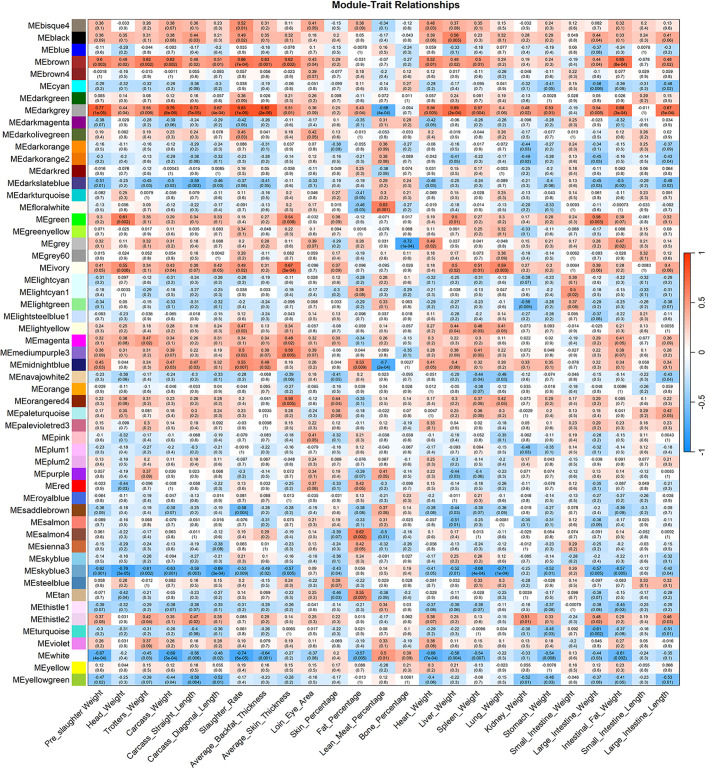
Construction of module-trait relationships matrix. Modules, created from WGCNA network with correlations (|R|) > 0.5 and q < 0.05, were used to construct the module-trait relationship plot using Pearson correlation. The darkgrey module exhibited a strong correlation (|R|) > 0.7 with important carcass traits, including pre-slaughter weight, slaughter rate, carcass weight, average backfat thickness and carcass straight length. This module may contain metabolite biomarkers linked to growth performance and fat deposition in Ningxiang pigs.

To further explore the biological relevance of metabolite changes, WGCNA was performed. A cut-off of R^2^ = 0.9 was used to determine the soft-thresholding power (β), and β = 9 was chosen for co-abundance network construction (Fig 6A). Metabolites exhibiting similar abundance patterns across developmental stages were clustered into distinct color-coded modules, whereas metabolites lacking clear co-abundance patterns were assigned to grey module. In total, 37 modules were constructed (Fig 6B) and subsequently integrated with previously published carcass traits [30] to construct module–trait relationship networks (Fig 7).

**Fig 8:**
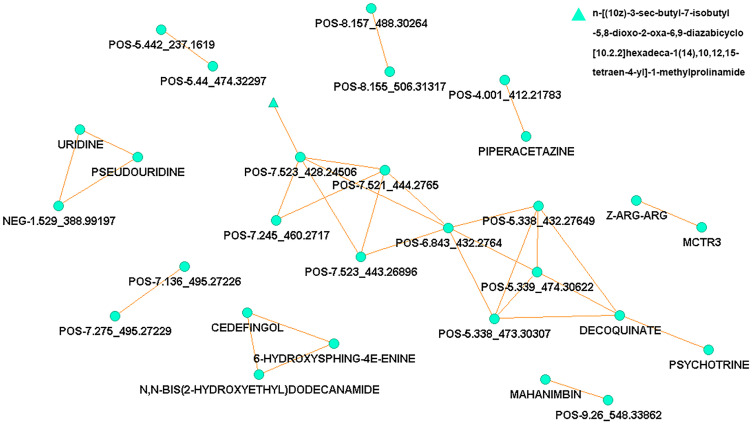
Co-abundance network with the hub metabolite psychotrine. Twenty-nine metabolites were found in this co-abundance network with 12 annotated metabolites distributed in 9 subnetworks. The central role of psychotrine in this network suggested that it may have acted as a key regulatory metabolite, potentially influencing multiple metabolic pathways during growth performance and fat deposition.

Hub metabolites (Top 5) from each module were exported from co-abundance network analysis. Among these, 13 hub metabolites were further prioritized based on their prior identification and significant associations between module membership and carcass traits (|R| > 0.5 and P-vaule<0.05). One of the major hub metabolites identified within the darkgrey module was psychotrine, which exhibited a progressive increase in backfat tissue across developmental stages in Ningxiang pigs. In addition, the darkgrey module-traits relationship exhibited strong positive correlation (R > 0.7) with important carcass traits, including pre-slaughter weight, slaughter rate, carcass weight, average backfat thickness and carcass straight length (File S5). Based on these findings, we constructed a co-abundance network with metabolites in darkgrey and identified 12 annotated metabolites that potentially associated with growth performance and fat deposition (Fig 8).

## Discussion

**Fig 9:**
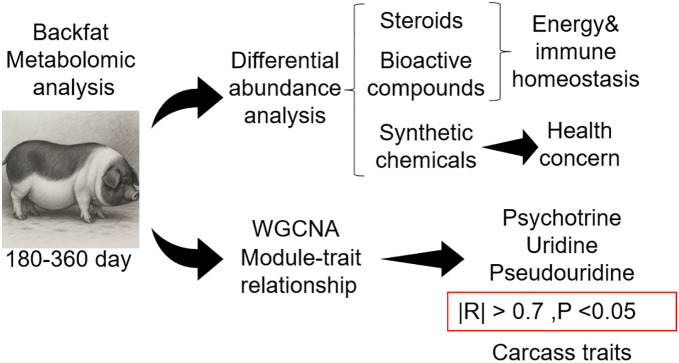
A summary diagram illustrated the key findings from the metabolomic analysis of Ningxiang pigs from 180d-360d. Differential abundance analysis revealed a dynamic profile of metabolites involved in energy regulation and immune homeostasis, alongside the accumulation of synthetic compounds potentially hazardous to animal and consumer health. The module-trait correlation network identified key metabolites, including psychotrine, uridine and pseudouridine, that strongly associated with economically vital carcass traits: pre-slaughter weight, slaughter rate, carcass weight, average backfat thickness and carcass straight length.

This study provides a comprehensive characterization of the backfat metabolome in Ningxiang pigs across key developmental stages (180–360 days), revealing substantial metabolic alterations associated with growth and fattening processes. The findings demonstrated distinct accumulation patterns of lipid, bioactive, and synthetic metabolites, highlighting the multifaceted role of adipose tissue not only as an energy storage organ but also as an active regulator of metabolic homeostasis. Additionally, network analysis identified several metabolites, including psychotrine, uridine, and pseudouridine, as potential biomarkers associated with economically important carcass traits, suggesting their potential value in breeding selection and production management strategies (Fig 9).

### Adipose tissue may function as a reservoir for lipid-soluble metabolites associated with immunometabolic regulation

In this study, we identified a variety of lipid-soluble compounds in the backfat of Ningxiang pigs, including endogenous lipids and exogenous bioactive metabolites, some of which are known to play critical roles in appetite regulation, oxidative stress, and inflammation.

Among the identified metabolites, methanandamide phosphate and oleoylethanolamide, are two important lipid mediators with opposing regulatory effects on appetite and energy homeostasis [33]. Methanandamide phosphate, an analog of the endocannabinoid anandamide [34, 35], acts via cannabinoid receptors to stimulate appetite and increase food intake [36–38]. In contrast, oleoylethanolamide is a potent appetite suppressant that reduces food intake and body weight [39–42]. It binds to peroxisome proliferator-activated receptor α to suppress hunger signals and promote the breakdown of fats [43–45]. In this study, methanandamide phosphate abundance decreased at 240 days, whereas oleoylethanolamide reached its highest level at 300 days, indicating a potential metabolic shift toward appetite suppression and enhanced lipid utilization during the fattening stage. This pattern may partially explain the relatively high feed conversion efficiency previously reported in Ningxiang pigs during later developmental stages [3].

Our metabolomic analysis also revealed the accumulation of several exogenous bioactive compounds in backfat, further supporting the role of adipose tissue as a metabolic interface linking nutrient storage and physiological regulation. For example, α-tocotrienol, a neuroprotective form of vitamin E, was elevated from 180 to 360 days. This accumulation may reflect enhanced antioxidant defense capacity during adipose tissue expansion, thereby protecting lipid-rich tissues from oxidative damage associated with increased fat deposition [46, 47]. Another compound, cephaeline, an isoquinoline alkaloid with reported antiviral and anti-cancer properties in mammals such as cats and rabbits [48–51], also accumulated with age. This accumulation may indicate age-related exposure, selective retention by adipose tissue, or a physiological role in immune regulation. The enrichment of these bioactive molecules suggests that backfat may act as a reservoir for bioactive compounds capable of influencing systemic metabolic and immune responses.

In contrast, several metabolites with known biological activities exhibited decreasing abundance during the fattening period, potentially reflecting reduced dietary exposure, altered gut microbial metabolism, or enhanced metabolic clearance with age. For instance, 2-phytyl-1,4-naphthoquinone, a biosynthetic precursor of vitamin K1, plays a role in coagulation and vascular function [52–54]. Its decline may indicate altered vitamin metabolism as pigs mature. Aurachin D, a bacterial-derived alkaloid with antimicrobial activity [55–57], and sanguinarine, a benzophenanthridine alkaloid with broad anti-inflammatory and anticancer effects [58–61], were both decreased, suggesting reduced microbial-derived compound absorption or a modulation of the immune-metabolic interface during adipose tissue expansion.

Another metabolite of interest was flazin, a cytoprotective β-carboline alkaloid capable of activating the KEAP1-NRF2 antioxidant signaling pathway, which plays a central role in cellular antioxidant defense [62]. The observed reduction in flazin abundance during development may indicate decreased oxidative stress or reduced reliance on antioxidant compensation mechanisms as adipose tissue matures. Similarly, pentosidine, a biomarker for advanced glycation end products [63], is associated with oxidative stress [64], insulin resistance [65], obesity [66], and chronic inflammation [67, 68]. Pentosidine abundance gradually declined from 180 to 360 days, suggesting a potential alleviation of oxidative stress and inflammatory burden during the fattening stage.

Collectively, these findings demonstrate that the temporal alterations in metabolite abundance reflect physiological adaptations associated with growth, nutrient utilization, and systemic metabolic regulation, underscoring the relevance of adipose tissue in maintaining immunometabolic balance.

### Accumulation of synthetic lipid-soluble compounds in backfat may present a potential health concern

The accumulation of synthetic lipid-soluble compounds in the backfat of Ningxiang pigs highlights a potential interface between environmental exposure and animal metabolism, with possible implications for pig health, breeding management, and pork safety. Our metabolomic analysis identified 21 synthetic metabolites, of which 14 exhibited progressively accumulation across developmental stages. These metabolites likely originate from environmental exposure, contaminated feed, or veterinary drug residues.

Decoquinate, for example, an anticoccidial agent widely used in livestock production, was detected in backfat tissue, and its accumulation may indicate prolonged or excessive exposure during the production cycle [69, 70]. Several detected contaminants were pharmaceutical compounds that may have been ingested through contaminated water. These include: (±)-methoprene, a juvenile hormone analog commonly applied as an insecticide [71, 72]. forasartan (SC-52458), an antihypertensive drug [73, 74]; galeterone, a steroidal antiandrogen used in prostate cancer treatment [75, 76]; testosterone decanoate, a synthetic androgen [77, 78]; allylestrenol, a synthetic progestogen used for preventing premature labor and recurrent miscarriage [79]; and piperacetazine, an antipsychotic prodrug used for treating schizophrenia and other psychotic disorders [80]. Although these compounds are not intended for application in swine production, their presence in adipose tissue implies bioaccumulation resulting from environmental contamination. Their presence also highlights the need for stricter monitoring and regulatory control of feed and water quality within livestock production systems.

In addition, two mycotoxin derivatives, hydrolyzed fumonisin B1 (HFB1) and paspaline, were identified in backfat tissue, suggesting contamination through improperly stored or mold- contaminated feed ingredients. Mycotoxins are known to cause immunosuppression, hepatotoxicity, and growth impairment in pigs and may also present residual risks to consumers through contaminated pork products [81].

Although the detected levels of individual synthetic compounds may not exceed established safety limits, their cumulative presence and potential synergistic effects raise concerns about long-term exposure. From a breeding perspective, our findings suggest that selection for pigs with enhanced metabolic clearance or lower xenobiotic accumulation in fat could improve carcass safety and health resilience. Moreover, from a food safety perspective, the presence of lipophilic contaminants in edible fat tissues underscores the need for stricter feed and water quality control, responsible drug management, and routine monitoring of synthetic residues in livestock. These results collectively emphasize the urgency of integrating metabolomic screening into breeding and farming practices to safeguard animal welfare and minimize downstream risks to public health.

### Integration of module-trait correlation analysis identifies candidate metabolites associated with carcass traits

To uncover metabolites potentially linked to economically important carcass traits, WGCNA was applied to construct a module-trait correlation matrix using metabolomic data. We found a key module, darkgrey, that exhibited a strong positive correlation (|R| > 0.7) with important carcass traits, including pre-slaughter weight, slaughter rate, carcass weight, average backfat thickness and carcass straight length. The hub metabolite in this module was psychotrine, which showed strong network connectivity and was directly linked to decoquinate, an antiprotozoal agent that has been previously associated with healthy growth and weight gain in livestock [82]. Psychotrine and cephaeline are both alkaloids of the emetine class, found in the ipecac root [83]. Preliminary evidence suggests that psychotrine dihydrogen oxalate may exhibit antiviral properties, including potential activity against human immunodeficiency virus [84, 85] and SARS-CoV-2 [86]. However, its physiological role in livestock remains entirely uncharacterized. Notably, our study is the first to implicate psychotrine in animal growth, identifying it as a hub metabolite in a network module strongly associated with economically important carcass traits. Its co-occurrence with decoquinate within the same metabolite network suggests a previously unrecognized metabolic association that warrants further investigation. These findings indicate that psychotrine may represent a novel candidate metabolite potentially involved in swine growth and fat deposition.

Interestingly, this module also included uridine and pseudouridine, both of which were positively correlated with important carcass traits. Uridine, a pyrimidine nucleotide essential for RNA synthesis [87], has been implicated in regulating food intake [88], neuroprotection [89], lipid metabolism [90, 91], and mitochondrial function [92]. Pseudouridine, a structural isomer of uridine introduced post-transcriptionally into RNA, enhances RNA stability and translational efficiency [93]. While direct evidence linking pseudouridine to energy homeostasis is limited, but its synthesizing enzyme, pseudouridine synthase, has been shown to regulate mitochondrial protein synthesis and function [94–96]. Therefore, the observed variation in pseudouridine abundance may reflect alterations in cellular energy metabolism associated with developmental progression or nutritional status.

Other metabolites within the darkgrey module, such as MCTR3 (a pro-resolving lipid mediator), mahanimbin (a plant-derived carbazole alkaloid), and piperacetazine (a pharmaceutical compound), were also positively correlated with carcass traits. However, their biological roles in fat deposition or energy metabolism in pigs remain largely unexplored. The inclusion of such diverse compounds in the same network highlights the complex and multifactorial nature of metabolic regulation during pig development.

Together, these results demonstrate that metabolite co-abundance patterns can reveal biologically meaningful relationships with carcass performance, offering candidate biomarkers for further functional validation and potential targets for nutritional regulation strategies or precision breeding in swine production.

## Conclusion

This study provides a comprehensive metabolomic characterization of backfat tissue in Ningxiang pigs across multiple developmental stages, uncovering stage-specific accumulation patterns of both endogenous bioactive and exogenous synthetic compounds. Notably, we identified metabolite co-expression networks strongly associated with economically important carcass traits, with psychotrine emerging as a novel candidate biomarker despite its previously undocumented role in livestock. In addition, the detection of lipophilic synthetic contaminants in adipose tissue underscores the importance of strengthened environmental management and feed quality surveillance in swine production systems. Together, these findings expand the current understanding of adipose tissue metabolism in indigenous pig breeds and provide valuable insights for improving meat quality, enhancing feed safety, and developing precision breeding strategies.

## Supporting information

S1 Fig Distribution of relative standard deviation values for all 3913 detected features. (TIF)

S2 Fig Principal component analysis of QC injections. (TIF)

S3 Fig Total ion current evaluation of QC injections. (TIF)

S4 Fig Distribution of differentially abundant mycotoxin derivatives in the backfat of Ningxiang pigs across 4 developmental stages. Different letters (a, b) above the boxes indicate statistical significance **(n = 6)**.
(TIF)

S1 Table Ingredient composition and calculated nutrient content of the experimental diet. (DOCX)

S1 File Results of ANOVA and Tukey’s HSD tests. (XLSX)

S2 File Parameters and settings used for weighted gene co-expression network analysis (WGCNA). (XLSX)

S3 File Module assignment of metabolites identified by WGCNA. (XLSX)

S4 File Carcass trait dataset used for module–trait correlation analysis. (XLSX)

S5 File Complete module–trait correlation matrix. (XLSX)

S6 File Untargeted metabolomic profiling data. (XLSX)

S7 File Functional annotation of identified metabolites. (XLSX)
