# Supplementary figures and images for: Metabolomic profiling of backfat in Ningxiang pigs reveals lipid dynamics and carcass trait associations during the fattening stage
Source: PLoS One. 2026 Jul 22;21(7):e0353743. doi: 10.1371/journal.pone.0353743 (PMC13390868; doi:10.1371/journal.pone.0353743)

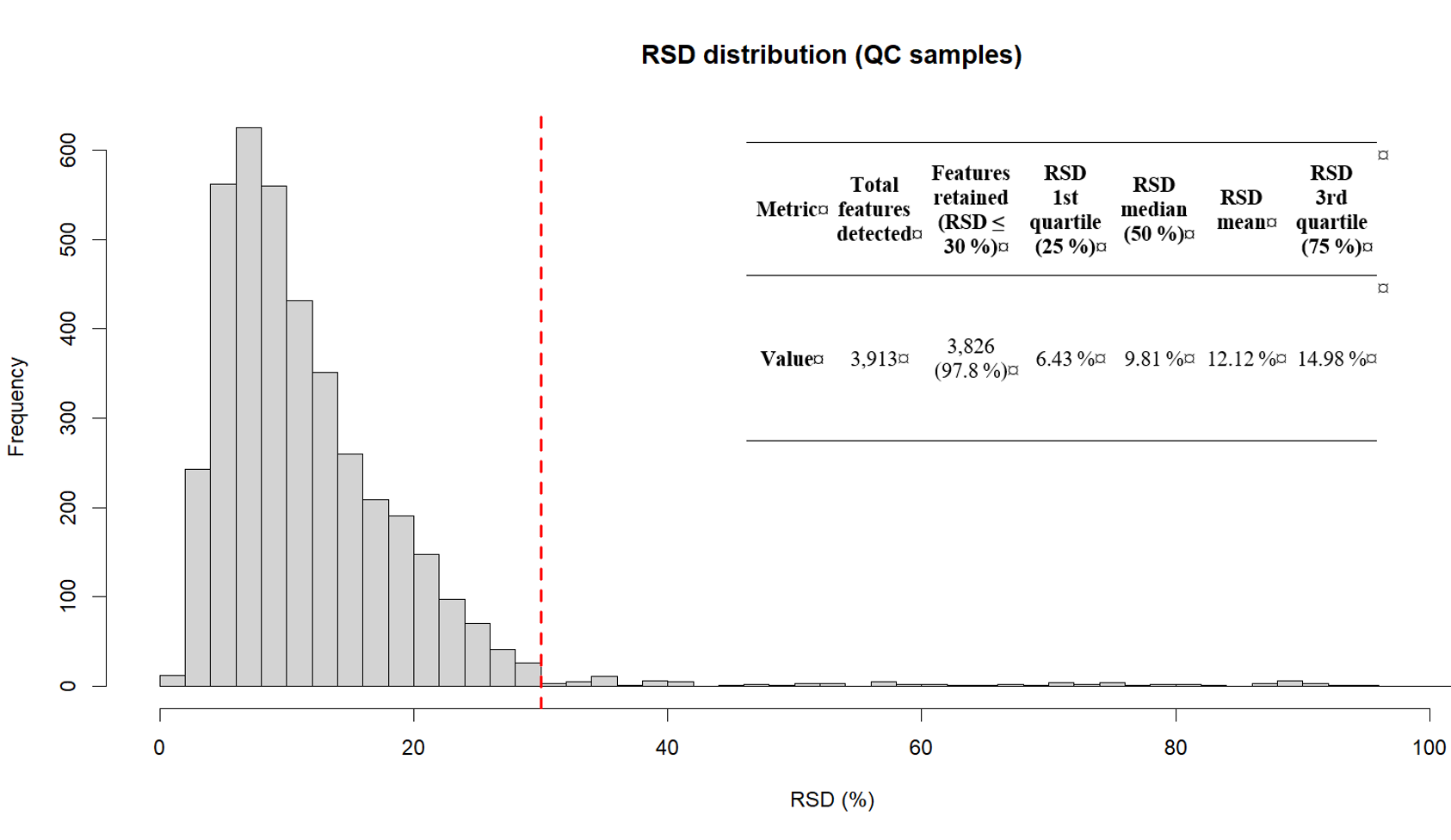

Supplement: S1 Fig — (TIF) [file pone.0353743.s001.tif]

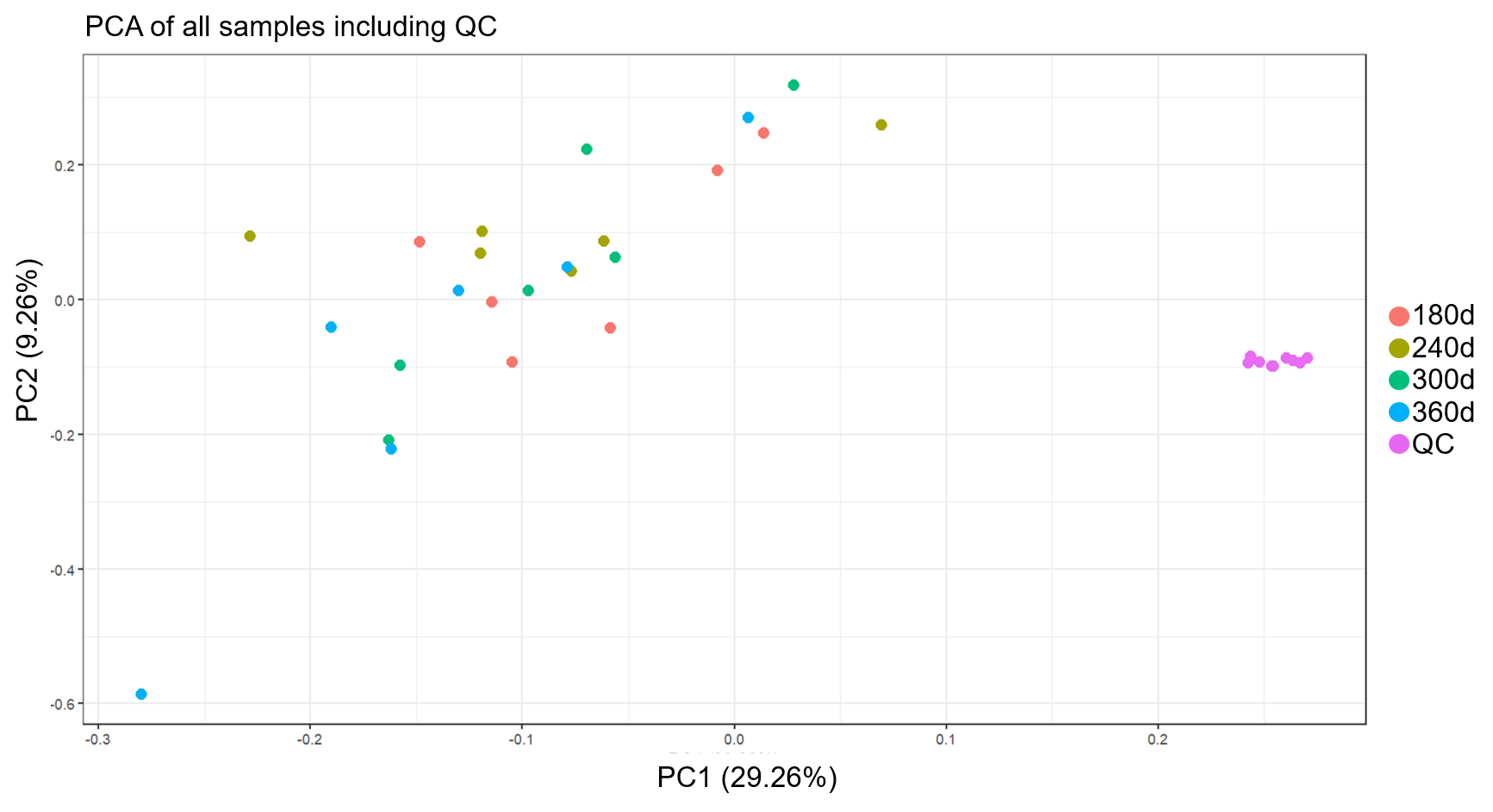

Supplement: S2 Fig — (TIF) [file pone.0353743.s002.tif]

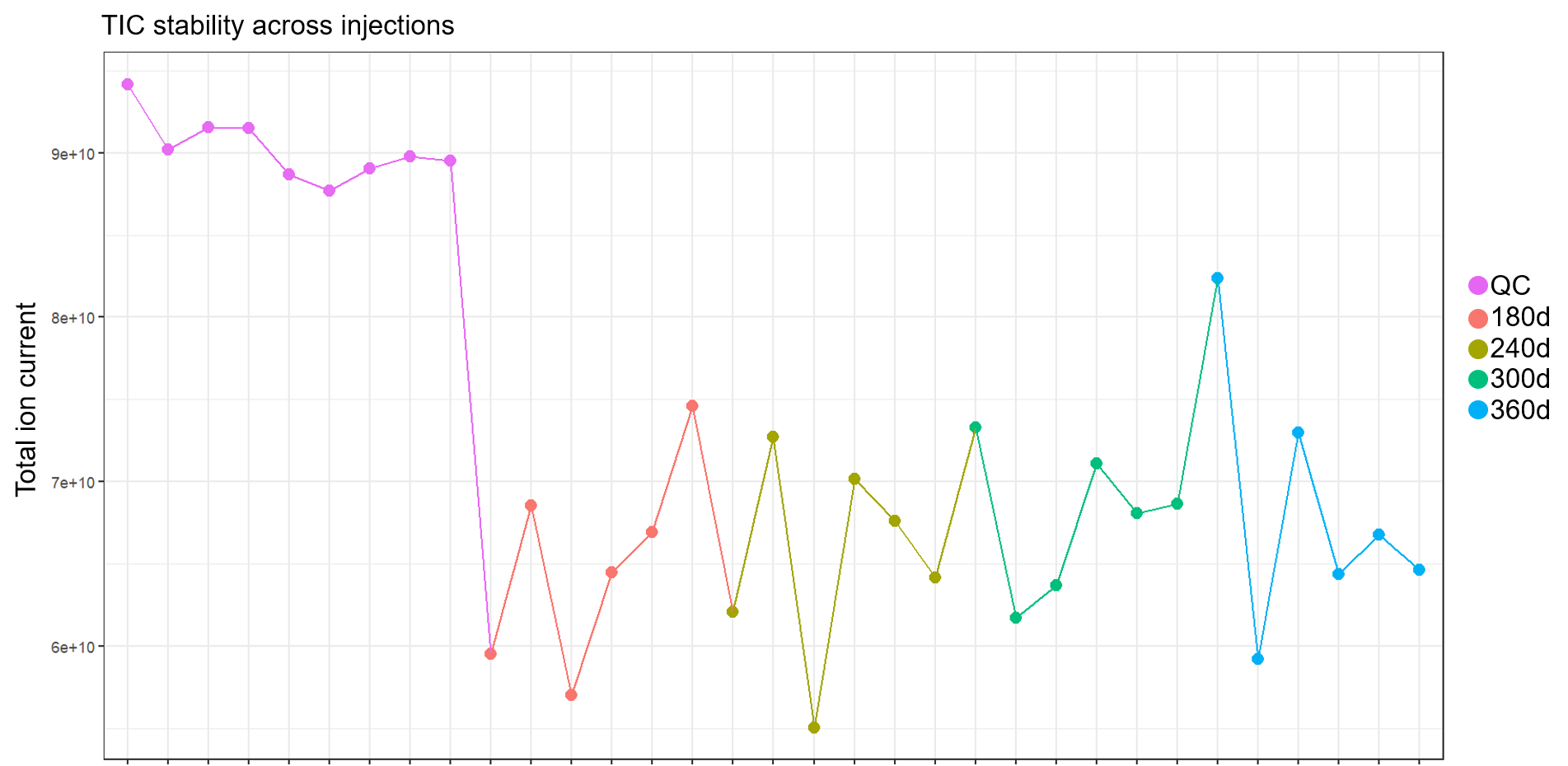

Supplement: S3 Fig — (TIF) [file pone.0353743.s003.tif]

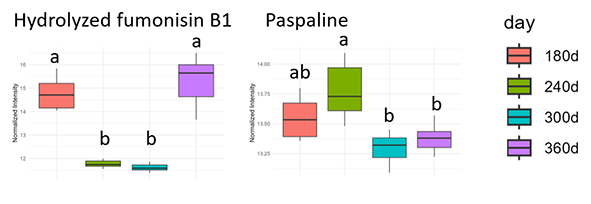

Supplement: S4 Fig — Different letters (a, b) above the boxes indicate statistical significance (n = 6). (TIF) [file pone.0353743.s004.tif]
