# Supplementary material for: Metabolomic profiling of backfat in Ningxiang pigs reveals lipid dynamics and carcass trait associations during the fattening stage
Source: PLoS One. 2026 Jul 22;21(7):e0353743. doi: 10.1371/journal.pone.0353743 (PMC13390868; doi:10.1371/journal.pone.0353743)
Supplement: S1 Table — (DOCX) [file pone.0353743.s005.docx]

Supplementary Table S1. Ingredient composition and calculated nutrient content of the experimental diet.

| **Ingredient** | **Inclusion (g/kg feed)** |
| --- | --- |
| Corn | 283 |
| Wheat | 150 |
| Wheat flour (Grade 1) | 100 |
| Wheat bran byproduct (3.9% ash) | 50 |
| Domestic DDGS (26% protein, 8% fat) | 30 |
| Soybean meal (43% CP) | 126 |
| Broken rice bran (9.0% CP) | 100 |
| Full-fat rice bran (14% fat) | 75 |
| Wheat bran (15% inclusion) | 43 |
| Soybean oil | 3 |
| Premix^a^ | 40 |
| **Total** | **1000** |
| **Nutrient Composition** |  |
| Digestible energy (kcal/kg)^b^ | 3150 |
| Net energy (kcal/kg) ^b^ | 2250 |
| Crude protein (%)^b^ | 15.87 |
| Crude fat (%)^b^ | 3.4 |
| Crude fiber (%)^b^ | 3.89 |
| Calcium (%)^b^ | 0.65 |
| Total phosphorus (%)^b^ | 0.6 |
| Available phosphorus (%)^b^ | 0.22 |
| Lysine (%)^b^ | 1.05 |

^a^ Purchased from Hunan Lifeng Biotechnology Co., Ltd (Changsha, China). Supplied per kilogram of diet: 19.8 mg CuSO4.5 H2O; 0.20 mg KI; 400 mg FeSO4.7 H2O; 0.56 mg NaSeO3; 359 mg ZnSO4.7 H2O; 10.2 mg MnSO4⋅H2O; 5 mg vitamin K (menadione); 2 mg vitamin B1; 15 mg vitamin B2; 30 μg vitamin B12; 135 μg vitamin A; 2.75 μg vitamin D3; 0.45 μg vitamin E; 80 mg choline chloride.

^b^ Values were estimated according to feed ingredient composition based on NRC (2012).
