# Supplementary material for: Metabolomic profiling of backfat in Ningxiang pigs reveals lipid dynamics and carcass trait associations during the fattening stage
Source: PLoS One. 2026 Jul 22;21(7):e0353743. doi: 10.1371/journal.pone.0353743 (PMC13390868; doi:10.1371/journal.pone.0353743)
Supplement: Attachment — Submitted filename: Response to Reviewers.docx [file pone.0353743.s014.docx]

PONE-D-25-26633

Metabolomic profiling of back fat in Ningxiang pigs: insights into lipid metabolism and carcass trait associations across later developmental stages

PLOS ONE

Dear Dr. Chen,

Thank you for submitting your manuscript to PLOS ONE. After careful consideration, we feel that it has merit but does not fully meet PLOS ONE’s publication criteria as it currently stands. Therefore, we invite you to submit a revised version of the manuscript that addresses the points raised during the review process.

*Thank you for your valuable comments and suggestions. We sincerely appreciate your time and effort in reviewing our manuscript. We have carefully revised the manuscript and addressed each of the issues point by point below.*

I have found grammatical inconsistencies multiple times in abstract section for example "Ningxiang pigs is a..." that should be "Ningxiang pigs are a...".

Your abstract is overpacked and you have provided too much technical detail for an abstract, making it harder for readers to quickly grasp the study's significance.

There is also lack of specificity in outcome interpretation for example the role of the bioactive and synthetic compounds isn't clearly contextualized.

I would suggest you to simplify sentences and clarify the findings for example “Twelve DAMs were classified as bioactive compounds…”that should be “Twelve bioactive compounds were significantly altered, with three increasing and nine decreasing across developmental stages.”

*We have corrected all grammatical errors, including the specific example mentioned. The abstract has been substantially streamlined to focus on the most essential information. We have clarified the biological and practical significance of the key findings, particularly regarding the roles of bioactive and synthetic metabolites. Also, we have revised sentences throughout the entire abstract to improve flow and precision.*

In introduction section, I have found redundancy in your text. Several statements repeat ideas (e.g., flavor and fatty acid uniqueness are discussed in multiple ways) multiple time.

You introduction section lack hypothesis, for example the rationale behind selecting specific developmental stages isn't clearly stated in introduction section. I would like to suggest you to revise the introduction section and clearly state the hypothesis at the end of the introduction.
“We hypothesize that distinct metabolic profiles in back fat develop over time and correlate with key carcass traits.”

*We have carefully revised the Introduction to eliminate repetitive statements, particularly those related to fat flavor and fatty acid composition. We agree that a clear hypothesis strengthens the focus of the study. As suggested, we have now explicitly stated the rationale for selecting the fattening stage (180–360 days) and included the hypothesis at the end of the Introduction.* *The revised statement reads:* “*We hypothesize that developmental stage-specific changes in the back fat metabolome of Ningxiang pigs reflect coordinated regulation of energy metabolism, and key metabolites within this profile are associated with economically important carcass traits and breeding performance.”*

In M and M section, I have found that there is insufficient detail in WGCNA setup: Module merging criteria and method validation are not discussed.

Furthermore, ethical statement buried in a paragraph: Hard to locate. Please add a brief validation comment on the WGCNA module significance (e.g., permutation testing) and format the ethical statement as a standalone subsection.

*We have added the detail in WGCNA setup to corresponding locations in methods:*

*“Modules were merged based on eigengene similarity using a cut height of 0.25 (correlation > 0.75). The biological relevance of modules was validated by correlating eigengenes with developmental stages. “*

*“Permutation testing (n = 1000) confirmed that significant module–trait associations were unlikely to occur by chance (p<0.05), supporting the robustness of the identified modules.”*

*We agree that the ethical approval details were not prominently displayed. We have now reformatted the ethical statement into a clearly labeled, standalone subsection titled* ***“Animal ethics”*** *to improve readability and compliance with standard reporting practices.*

In result section, you overly descriptive with little interpretation: For example, “Fig 1 shows PC1 and PC2...” is descriptive without biological insight

you used inconsistent terminology several time for example Sometimes “DAMs,” other times “metabolites” or “chemicals.” I suggest you to add brief biological implications under each figure for example “These lipids may contribute to the increased fat deposition observed at later stages.” And don’t forget to use consistent terminology (DAMs/metabolites).

*We agree that parts of the Results section were overly descriptive and lacked sufficient biological interpretation. In response, we have added the relevant sections to include more insight into the biological significance of the findings. We have replaced “DAMs” and chemicals with metabolites to keep the consistency. In addition, we have added brief biological implications under each figure.*

In discussion section, similar information appears in the introduction and discussion. And authors failed to fully explore the results in this section. Authors provided limited integration of findings and discussion does not fully connect WGCNA and metabolite trends to pig physiology. I suggest you to synthesize findings instead of listing them for example, “The increased abundance of oleoylethanolamide at 300d suggests a possible regulatory shift toward reduced feed intake, potentially explaining the observed feed efficiency at later stages.”

*We have removed repetitive background information from the discussion that was already covered in the introduction. We restructured the discussion to better integrate and interpret the metabolite trends in the context of pig physiology. Rather than listing individual metabolite changes, we now emphasize overarching patterns, such as temporal shifts in lipid and bioactive compounds, and their biological implications, including appetite regulation, oxidative stress, etc. Additionally, we strengthened the connection between WGCNA modules and carcass traits by contextualizing hub metabolites (e.g., psychotrine, uridine, pseudouridine) with known physiological functions such as energy metabolism and mitochondrial regulation.*

Conclusion is too vague to understand and the conclusion doesn’t strongly restate the importance or novelty of the work.

*We have rewritten the conclusion to be more specific and impactful.*

I have found grammar and syntax errors throughout the manuscript. Authors used inconsistent use of past and present tense. I suggest authors for thorough proofreading by a native English speaker or professional editing service. Please maintain past tense for describing methods and results, present tense for general knowledge.

*We have used a professional editing tool to correct grammatical errors throughout the entire manuscript. In addition, we would like to clarify that, in accordance with standard scientific writing conventions, we have used the* ***past tense*** *when describing our experimental design in the* ***Introduction*** *and when discussing our findings in the* ***Discussion section.***

Journal requirements:

When submitting your revision, we need you to address these additional requirements.

1. Please ensure that your manuscript meets PLOS ONE's style requirements, including those for file naming. The PLOS ONE style templates can be found at

<https://journals.plos.org/plosone/s/file?id=wjVg/PLOSOne_formatting_sample_main_body.pdf> and

<https://journals.plos.org/plosone/s/file?id=ba62/PLOSOne_formatting_sample_title_authors_affiliations.pdf>

*We have ensured that our manuscript meets the journal style requirements.*

2. To comply with PLOS ONE submissions requirements, in your Methods section, please provide additional information regarding the experiments involving animals and ensure you have included details on (1) methods of sacrifice, (2) methods of anesthesia and/or analgesia, and (3) efforts to alleviate suffering.

*We have included additional information regarding the animal handling and ethics in methods section: “The pigs were provided with free access to clean water and a nutritionally balanced diet throughout the study. They were monitored daily for signs of illness or distress, and any sick or injured animals received prompt veterinary care in accordance with institutional guidelines. The pigs were humanely euthanized using electrical stunning followed by slaughter.”*

3. Please note that PLOS ONE has specific guidelines on code sharing for submissions in which author-generated code underpins the findings in the manuscript. In these cases, we expect all author-generated code to be made available without restrictions upon publication of the work. Please review our guidelines at <https://journals.plos.org/plosone/s/materials-and-software-sharing#loc-sharing-code> and ensure that your code is shared in a way that follows best practice and facilitates reproducibility and reuse.

*We have uploaded our R code on protocols.io,* *http:// dx.doi.org/10.17504/protocols.io.kqdg3wj51v25/v1, and added the doi link to the method section.*

4. Thank you for uploading your study's underlying data set. Unfortunately, the repository you have noted in your Data Availability statement does not qualify as an acceptable data repository according to PLOS's standards.

At this time, please upload the minimal data set necessary to replicate your study's findings to a stable, public repository (such as figshare or Dryad) and provide us with the relevant URLs, DOIs, or accession numbers that may be used to access these data. For a list of recommended repositories and additional information on PLOS standards for data deposition, please see <https://journals.plos.org/plosone/s/recommended-repositories>.

*We have revised Data Availability statement.*

The raw data will be available upon publication: Figshare: 10.6084/m9.figshare.29361956.

For reviewers to access, please use private link: https://figshare.com/s/2c28cc686518afd040b2

5. We note that Figure 9 in your submission contain copyrighted images. All PLOS content is published under the Creative Commons Attribution License (CC BY 4.0), which means that the manuscript, images, and Supporting Information files will be freely available online, and any third party is permitted to access, download, copy, distribute, and use these materials in any way, even commercially, with proper attribution. For more information, see our copyright guidelines: <http://journals.plos.org/plosone/s/licenses-and-copyright>.

We require you to either (1) present written permission from the copyright holder to publish these figures specifically under the CC BY 4.0 license, or (2) remove the figures from your submission:

*We have replaced the image in Fig 9 with a new version that does not have any copyright issues.*

6. We note that there is identifying data in the Supporting Information file <Supplementary Material S1.xlsx>. Due to the inclusion of these potentially identifying data, we have removed this file from your file inventory. Prior to sharing human research participant data, authors should consult with an ethics committee to ensure data are shared in accordance with participant consent and all applicable local laws.

*We would like to clarify that our study did* ***not*** *involve human research participants. The data provided in Supplementary Material S1.xlsx are exclusively derived from* ***pigs*** *used in the animal experiment.*

*We have reviewed the spreadsheet and can confirm that it does* ***not*** *contain any personally identifiable information or sensitive data related to humans. All identifiers in the file pertain solely to animals and are used for tracking purposes within the study (e.g., animal stage), which do not pose any privacy or ethical concerns.*

*We will upload the file again if it is needed.*
