# Supplementary material for: Metabolomic profiling of backfat in Ningxiang pigs reveals lipid dynamics and carcass trait associations during the fattening stage
Source: PLoS One. 2026 Jul 22;21(7):e0353743. doi: 10.1371/journal.pone.0353743 (PMC13390868; doi:10.1371/journal.pone.0353743)
Supplement: Attachment — Submitted filename: Response_to_Reviewers_auresp_2.docx [file pone.0353743.s016.docx]

Additional Editor Comments:

Dear Authors
The revised manuscript has addressed several of the initial concerns, but significant issues remain that affect the overall quality and clarity of the work. Below are the key gaps and problems that warrant rejection:

The abstract is still overly dense and lacks proper structure. It should be concise and formatted as a single paragraph. Statistical descriptions (e.g., p-values, effect sizes) are missing, which are critical for interpreting the significance of the findings. The abstract should clearly state the novelty and broader implications of the study, which are currently unclear.

*We have revised the abstract to ensure it is concise, clearly structured as a single paragraph, and free from unnecessary density. P-values have been added to highlight the significance of our key findings. We also clarified the novelty and broader implications of the study to better communicate its scientific contribution.*

I have also found inconsistent terminologies throughout the manuscript for example manuscript still uses terms like "DAMs" (differentially abundant metabolites) interchangeably with "metabolites" or "chemicals," which can confuse readers.

*We agree that using terms like "DAMs," "metabolites," and "chemicals" interchangeably may cause confusion for readers. In response, we have carefully reviewed the entire manuscript and* ***removed all instances of “DAMs” and other inconsistent or unclear terminology****. We now consistently use the term* ***“metabolites”*** *throughout the text and figure labels to maintain clarity and uniformity. These revisions have been applied in the main text and figure legends as appropriate.*

Moreover, the results section remains overly descriptive without sufficient biological context. For example, the PCA analysis (Fig 1) is described but not linked to meaningful biological shifts in fat metabolism.

*We have revised and linked PCA to meaningful biological shifts in our results: “The metabolite profile at 180d was clearly separated from those at later stages (240–360d), contributing most to the variation along PC1. This suggests a significant metabolic shift occurring after 180 days, likely reflecting the onset of the fattening phase, where energy storage and lipid biosynthesis become more prominent.” Please note that* ***PCA itself does not directly show biological processes****, but only suggest when major biological shifts might occur.*

*The main purpose of PCA in this study was to explore overall variation in the metabolomic data and assess whether samples clustered by developmental stage. PCA is* ***exploratory****, not* ***confirmatory****. If PCA shows* ***clear stage-based separation****, it's reasonable to then* ***prioritize*** *those comparisons, but this decision should also be backed by following analysis.*

*We have also revised the rest part of results with sufficient biological context.*

Furthermore, statistical details (e.g., ANOVA results, post-hoc test outcomes) are either missing or buried in supplementary files. These should be highlighted in the main text.

*The statistical details, including ANOVA results and post-hoc test outcomes, were described in the “Statistics” section of the Methods and provided in full in the Supplementary Files due to the large number of pairwise comparisons across all metabolites. Given the scale of the data, it is not practical to list every statistical outcome in the main text or a table. Instead, we followed standard practice by visualizing key results using heatmaps and scatterplots with Compact Letter Display (CLD) to indicate statistically significant differences, as shown in Figures 2–5.*

*To address your concern and better highlight the significance of our findings, we have now:* ***Added p-values (e.g., p < 0.05) directly in the figure legends and main text*** *where appropriate, and ensured that* ***visual indicators of significance*** *(e.g., CLDs) are clearly described and linked to the underlying statistics.*

*We hope these changes improve the clarity and accessibility of the statistical results.*

The WGCNA analysis lacks clarity in how modules were validated or how hub metabolites were selected.

*These details were already described in both the Methods and Results sections, and we also uploaded our R scripts for clarity (as requested): The R code can be accessed from http:// dx.doi.org/10.17504/protocols.io.kqdg3wj51v25/v1.*

*To make sure that it has been sufficiently emphasized, we further revised the relevant text (in methods) to clarify the analytical steps:*

***Module validation:*** *To ensure robustness, we performed 1,000 permutation tests, confirming that the observed module–trait associations were unlikely to occur by chance (p < 0.05). Only modules with strong correlations (|R| > 0.5, p < 0.05) were included in downstream analysis.*

***Hub metabolite selection:*** *Within each trait-associated module, we ranked metabolites based on their module membership (MM) values. Metabolites with the highest MM values were considered "hub metabolites," as they represent highly connected nodes within the network. Among these, we further prioritized hub metabolites that also showed significant correlation with carcass traits (|R| > 0.5, p < 0.05), ensuring both topological and phenotypic relevance.*

The discussion fails to synthesize findings into a coherent narrative. For example, the role of psychotrine in carcass traits is mentioned but not contextualized within existing literature.

*Psychotrine is an underexplored metabolite, with only nine publications listed in PubMed and none addressing its role in animal growth or carcass traits. Our identification of psychotrine as the hub metabolite in a module strongly correlated with key carcass performance indicators—alongside decoquinate, a known growth-promoting agent—suggests a previously unrecognized link between this emetine-class alkaloid and livestock development. This co-occurrence within a growth-related metabolic network presents a novel finding that expands current understanding of carcass trait regulation and highlights psychotrine as a promising candidate for further functional investigation in swine physiology.*

*We have revised the discussion to emphasize the novelty of this finding, highlighting psychotrine as a previously uncharacterized metabolite potentially associated with growth-related traits in swine.*

The discussion of bioactive and synthetic compounds lacks depth.

*We have substantially revised the discussion to provide deeper insight into the biological relevance of both bioactive and synthetic compounds identified in back fat. Specifically, we expanded the interpretation of several key metabolites by discussing their known functional roles, sources, and potential physiological implications.*

The health risks of synthetic compounds are noted, but their practical implications for breeding or food safety are not discussed in depth.

*We have revised the discussion section to better articulate the practical implications of our findings.*

The implications for pig health or meat quality are not fully explored.

*In the revised discussion section, we have expanded on the implications of our findings for pig health and meat quality. Specifically, we discuss how shifts in metabolite profiles—such as increased antioxidant compounds (e.g., α-tocotrienol) and decreased pro-inflammatory or oxidative stress–related metabolites (e.g., pentosidine)—may indicate improved metabolic resilience and fat quality during the fattening period. Conversely, the accumulation of synthetic or potentially harmful exogenous compounds raises concerns regarding long-term animal health and pork safety. These findings offer valuable insights for optimizing feeding practices, improving meat quality traits, and informing breeding strategies aimed at enhancing metabolic efficiency and minimizing contaminant accumulation.*

At the end I would say that conclusion is vague and does not strongly restate the study's novelty or actionable insights. It should explicitly highlight how the findings advance the field.
*We have revised the conclusion to more clearly articulate the study's novelty and practical implications.*
