# Supplementary material for: Metabolomic profiling of backfat in Ningxiang pigs reveals lipid dynamics and carcass trait associations during the fattening stage
Source: PLoS One. 2026 Jul 22;21(7):e0353743. doi: 10.1371/journal.pone.0353743 (PMC13390868; doi:10.1371/journal.pone.0353743)
Supplement: Attachment — Submitted filename: PONE-D-25-26633_R2 Comments.docx [file pone.0353743.s015.docx]

The authors have addressed many reviewer questions, but important methodological, reporting and interpretive problems remain that prevent the paper from being acceptable in its present form.

**Abstract**

Lines 23-24: “A total of 154 metabolites exhibited significant temporal variation (p<0.05).”…. Please clarify whether the count of 154 metabolites refers to raw p-values (p < 0.05) or to FDR-adjusted q-values. Because untargeted metabolomics tests thousands of features, multiple testing correction is essential.

**Materials and Methods**

Lines 116-118: “A pooled quality control (QC) sample was injected after every 10 samples to monitor instrument stability and reproducibility during LC-MS analysis.”….. Please report QC metrics (e.g., PCA of QC clustering, RSD cutoff and % features removed, TIC stability, and features retained) in the main text or Supplement. If no RSD filter was applied, justify. These details are needed to support data quality claims.

Lines 163-164: “P-values were adjusted using the Benjamini-Hochberg correction.”…. The Methods state that p-values were adjusted using Benjamini–Hochberg, but the Results/Abstract report significance thresholds (p<0.05) without making clear whether quoted counts reflect adjusted q-values. Please revise all counts in the Abstract and Results so they explicitly say whether they are raw p or FDR q counts.

**Results**

Lines 170-171 and 188-190: “Untargeted metabolomic profiling identified 3,913 metabolites in back fat tissues of Ningxiang pigs across four developmental stages (180, 240, 300, and 360 days).” And “To identify temporally regulated metabolites and assess their functional properties, we next performed differential abundance analysis and identified 154 metabolites that significantly varied across the four developmental stages.”…………… Please distinguish between detected features (3,913) and annotated metabolites. For the 154 significant ones, indicate how many are high-confidence (MSI 1–2) vs. putative (MSI 3–4).

Lines 144-145 and 246-248: “WGCNA identified 37 different modules, each assigned with a distinct color, with highly correlated metabolites grouped together.” And “Thirty-six modules were 12 constructed (Fig 6B) and further used to build up module-trait relationships along with our previous published carcass traits [29] (Fig 7).”………..The manuscript inconsistently reports 36 vs. 37 modules. Please make this consistent, clarify whether the grey module was counted, and provide WGCNA parameters, module sizes, and the module–trait correlation matrix in the supplement.

Lines 146-147: “Our previously published carcass traits data were used to build up module-trait relationships [29].”….. Please clarify if carcass traits were measured on the same animals as the metabolomics samples. If yes, provide a table (IDs, trait values, normalization). If not, justify the use of external data and discuss the limitations of module–trait correlations.

Lines 208-214: “We also found that twenty-one metabolites were synthetic metabolites, with twelve of them accumulated from 160d to 360d, five increased at various stage, four gradually decreased in the back fat from 160d to 360d (Fig 5). In addition, we identified two mycotoxins, hydrolyzed fumonisin B1 (HFB1) and paspaline in the back fat. HFB1 was higher in the back fat at 160d and 360d, but paspaline was decreased from 240d (S1 File). The function of remaining metabolites was unknown due to lack of information in the database. Detailed information was listed in S1 File”……The Results section refers to 160d, but Abstract and Methods specify 180d as the earliest time point. Please correct these inconsistencies and ensure all text, figures, and supplements consistently use 180d, 240d, 300d, and 360d.

**References**

All references should be reformatted according to the journal’s reference style. Please ensure consistency in author listing, journal names, title capitalization, punctuation, and DOI formatting.
