# Supplementary material for: Metabolomic profiling of backfat in Ningxiang pigs reveals lipid dynamics and carcass trait associations during the fattening stage
Source: PLoS One. 2026 Jul 22;21(7):e0353743. doi: 10.1371/journal.pone.0353743 (PMC13390868; doi:10.1371/journal.pone.0353743)
Supplement: Attachment — Submitted filename: Response to reviewers Nov 12.docx [file pone.0353743.s018.docx]

Please improve methodology transparency, data consistency, and interpretation. Issues include unclear QC validation, inconsistent timepoints and module reporting, and insufficient explanation of statistical corrections and metabolite annotation. Additionally, language, formatting, and figure labeling need improvement to ensure clarity, accuracy, and compliance with journal standards.

The study presents a valuable metabolomic analysis of backfat development in Ningxiang pigs, but requires major revisions in methodology transparency, data consistency, and interpretation. Issues include unclear QC validation, inconsistent timepoints and module reporting, and insufficient explanation of statistical corrections and metabolite annotation. Additionally, language, formatting, and figure labeling need improvement to ensure clarity, accuracy, and compliance with journal standards.
**Response:**

We sincerely thank the reviewers and editors for their constructive feedback and valuable suggestions. In response, we have thoroughly revised the manuscript to improve **methodological transparency, data consistency, and interpretation**. The **QC validation** section has been expanded with detailed descriptions of feature filtering, reproducibility metrics, and instrument stability results. **WGCNA module information** have been carefully cross-checked to ensure internal consistency across the text, figures, and supplementary files. In addition, the **language, formatting, figure labeling, and supplementary materials** have been comprehensively edited to enhance clarity, accuracy, and compliance with journal standards. Point-by-point responses to all reviewer comments are provided below.

Reviewer #1:

(1) The typical slaughter age for Ningxiang pigs is 8 months. However, in the study, the pigs were raised until they reached 360 days old. Has this exceeded the standard slaughter age requirement?

**Response:**

We thank the reviewer for this important observation. While the typical commercial slaughter age for Ningxiang pigs is around 8 months (~240 days), the goal of our study was to investigate temporal changes in metabolomic profiles and backfat development across a broader developmental period, including late-finishing stages. Raising pigs to 360 days allowed us to capture full-body maturity and late-stage metabolic changes, which are critical for understanding fat deposition and quality traits. All procedures were conducted in strict accordance with animal welfare guidelines, and the extended rearing did not compromise the health or welfare of the animals.

(2) I think that the PCA plot only indicates that there are significant differences among the four stages, but it does not represent the conclusion described by authors.

**Response:**

We thank the reviewer for the comment. We agree that the PCA primarily shows overall differences in metabolomic profiles among developmental stages rather than providing mechanistic conclusions. Therefore, we have revised the text to more accurately reflect the descriptive nature of PCA.

(3) The result does not seem to cover all the classification results of non-targeted metabolites.

**Response:**

The results do cover all the classification results of non-targeted metabolites; however, this may not have been sufficiently clear. The same concern was also raised by Reviewer 2. To address this, we have now explicitly specified the identities of these metabolites in the Results section.

(4) If there are other references for using metabolites for co-expression analysis, please add them to the methods section.

**Response:**

There are many studies used metabolites for co-expression analysis, we have added a reliable reference to the methods section. The sentence reads “**Further details on metabolite-based co-expression analysis can be found in previous studies integrating metabolomic data for network construction [****32****].**”

Reviewer #2:

This study presents a metabolomic analysis of back fat in Ningxiang pigs across the fattening stage (180–360 days), a critical developmental window for lipid deposition and carcass quality. The work provided a metabolic atlas of backfat dynamics during this period in an indigenous pig breed. However, several issues require revision to improve clarity, accuracy, and rigor.

In abstract

1. Abbreviations in the text should be defined at their first appearance. For example, in the abstract (line: 30), 'weighted gene co-expression network' should be followed by their abbreviations. Additionally, 'weighted gene co-expression network' is missing the word 'analysis' (should be 'weighted gene co-expression network analysis (WGCNA). Similar issues may exist elsewhere; please conduct a thorough review of the entire manuscript.

**Response:**

We thank the reviewer for this helpful editorial comment. We have revised the manuscript to define all abbreviations at their first appearance and to correct wording errors.

2. Line 22–23: "metabolomic profile" should be changed to the plural form "profiles" because the study involves multiple time points.

**Response:**

Revised as suggested. The phrase now reads **“metabolomic profiles”** to reflect multiple sampling time points.

3. Line 24, the less-than sign (<) in (p<0.05) should be formatted with spaces around the operator, i.e., "(p < 0.05)", as shown on line 30. Please conduct a thorough check throughout the entire manuscript.

**Response:**

All instances of p-values have been checked and reformatted to include spaces, e.g., **(p < 0.05)**, consistently throughout the manuscript.

In the Keywords, please change Sus scrofa domesticus to Ningxiang pigs

**Response:**

The keyword **“Sus scrofa domesticus”** has been replaced with **“Ningxiang pigs.”**

In introduction

1. In line 49: The thickness of "backfat" is a critical indicator in pig breeding and meat production". The term "backfat" appears in two different forms throughout the manuscript: "backfat" and "back fat". Please standardize and use "backfat" consistently throughout the entire text.

**Response:**

All instances have been standardized to **“backfat.”**

Line 59: Ningxiang pigs, like many Chinese indigenous pig breeds, reach sexual maturity relatively early (120 day(d)), but full body maturity occurs later (300-360d). Please delete day ().

**Response:**

Revised as suggested. The sentence now reads:

**“Ningxiang pigs, like many Chinese indigenous pig breeds, reach sexual maturity relatively early (120 d), but full body maturity occurs later (300–360 d).”**

Line 77-78: The pigs were provided with free access to clean water and a nutritionally balanced diet throughout the study. Please change "provided with" to "had ad libitum access to".

**Response:**

The sentence has been revised to:

**“The pigs had ad libitum access to clean water and a nutritionally balanced diet throughout the study.”**

Lines 85-89: The manuscript should include a clear description of the slaughter method used. Specifically, please clarify whether electrical stunning or intravenous injection (e.g., pentobarbital) was employed for euthanasia.

**Response:**

We appreciate the reviewer’s attention to animal welfare details. The requested information is already included in the manuscript. The relevant sentence reads:

**“The pigs were humanely euthanized using electrical stunning followed by slaughter.”**

In Materials and Methods

Line 86：It is recommended to provide the general composition of the diet used in the study in a supplementary table.

**Response:**

We thank the reviewer for this valuable suggestion. We have now added a supplementary table (Table S1) providing the ingredient composition and calculated nutrient contents of the diet used in the method.

1. Line 132: The package "ggplot" should be corrected to "ggplot2", as "ggplot" is outdated and the current standard is "ggplot2". Additionally, please provide version numbers for all software and R packages used, such as ggplot2 and WGCNA, to ensure reproducibility. In addition, the statement "Detailed ANOVA and Tukey’s HSD test results were listed in the S1 File" could be improved. It is recommended to format the supplementary materials as clearly labeled tables (e.g., Table S1, Table S2, Table S3) to enhance readability and facilitate reference.

**Response:**

Revised as suggested. The package name has been corrected to **“ggplot2”**, and version numbers for all software and R packages (e.g., R v 4.4.2, ggplot2 v3.5.2, WGCNA v1.73) have been added to ensure reproducibility.

Additionally, the supplementary materials have been reformatted into clearly labeled supplementary tables (**Table S1..etc**) for improved clarity and reference. However, it is impractical to reformat and present some results into tables due to the large number of data included. These results are still presented as supplementary files (excel files).

2. In line 150: Please ensure consistent use of lowercase "p" for p value throughout the manuscript (e.g., p < 0.05).

**Response:**

All instances of statistical significance notation have been checked and standardized to use **lowercase “p”** consistently throughout the manuscript (e.g., **p < 0.05**).

In Results

1. In line 171: Untargeted metabolomic profiling identified 3,913 metabolites in back fat tissues of 171 Ningxiang pigs across four developmental stages (180, 240, 300, and 360 days). Please provide the total number of metabolites identified at each developmental stage, and indicate in the text which supplementary table (e.g., Table S1, S2, etc.) contains the detailed metabolite data.

**Response:**

In our study, untargeted LC–MS/MS analysis was performed under identical chromatographic and mass spectrometric conditions for all samples. Therefore, metabolite detection was based on a unified peak alignment and identification process across all developmental stages, resulting in a single set of 3,913 metabolites detected in the overall dataset. As such, the total number of metabolites identified does not differ among the four stages (180, 240, 300, and 360 days). However, the relative intensities of many metabolites varied significantly among stages, reflecting biological differences in metabolic activity during fat deposition.

2. Please include the percentage of variance explained by each principal component (e.g., PC1 and PC2) on the axes of the PCA plot in Fig 1

**Response:**

The percentage of variance explained by each principal component has now been added to the axes of the PCA plot in Figure 1 (PC1: 38.77%, PC2: 17.04%, PC3:10.37%).

3. There is an incorrect sequence in the statistical description in Lines 126–131. One-way ANOVA should first be used to test whether there are significant differences among all groups (overall significance). This is followed by P-value adjustment (e.g., Benjamini-Hochberg correction). Only for metabolites showing significant results in the ANOVA (after correction) should post-hoc pairwise comparisons (e.g., Tukey’s HSD test) be performed to identify which specific group pairs differ significantly. Additionally, the sentence "Boxplots of these metabolites were generated with ggplot2" should be placed after these analytical steps. Furthermore, the phrase "with basic R scripts" in line 127 is unnecessary and non-standard; it should be revised to simply state that analyses were performed using R.

**Response:**

The section has been revised to clearly describe the correct sequence of analyses: one-way ANOVA for overall significance, followed by Benjamini–Hochberg adjustment, and then Tukey’s HSD test for post-hoc comparisons. The redundant phrase “with basic R scripts” was removed, and the description of boxplot generation has been placed appropriately after the statistical steps.

4. Line 177 “With 3 metabolites being higher in back fat at 180d and 360d, five metabolites increased at various stages, and 6 metabolites decreased from 160d to 360d (Fig 3)”. However, the specific identities of these metabolites are not mentioned in the text. Please add the names of these metabolites in the manuscript to point out which metabolites you are mentioned. Alternatively, clearly label the metabolites in Figures 3, 4, and 5 (e.g., Fig 3 A-B or Fig 3 C-D), and refer to them accordingly in the main text to improve clarity and traceability.

**Response:**

We appreciate the reviewer’s suggestion to improve clarity. The manuscript and corresponding figures have been updated to specify the identities of the metabolites mentioned in Line 177. The metabolite names have been already labelled along with the bar chart in Fig 3-5.

5. In the figure legends of Figures 3, 4, and 5, the sample size annotation "(n=6)" should be placed at the end of the legend, rather than immediately after the title.

**Response:**

The sample size annotation “(n = 6)” has been moved to the end of the figure legends for Figures 3, 4, and 5 to improve consistency and readability.

In conclusion

1. Line 32 and 435 “This study provides the first comprehensive metabolomic profiling of back fat in Ningxiang pigs across developmental stages”. The use of "first" should be used with caution, as you have cited several previous studies on metabolomic profiling in pigs or related contexts. Please revise this claim in abstract and conclusion to ensure it is accurate and appropriately qualified.

**Response:**

The phrasing has been revised in both the Abstract and Conclusion to avoid overstatement.

Reviewer #3:

The authors have addressed many reviewer questions, but important methodological, reporting and interpretive problems remain that prevent the paper from being acceptable in its present form.

Abstract

Lines 23-24: “A total of 154 metabolites exhibited significant temporal variation (p<0.05).”…. Please clarify whether the count of 154 metabolites refers to raw p-values (p < 0.05) or to FDR-adjusted q-values. Because untargeted metabolomics tests thousands of features, multiple testing correction is essential.

**Response:**

We thank the reviewer for the comment. We used FDR-adjusted q-values (Benjamini–Hochberg correction) to identify significantly altered metabolites. To avoid confusion, all p < 0.05 have been changed to q < 0.05.

Materials and Methods

Lines 116-118: “A pooled quality control (QC) sample was injected after every 10 samples to monitor instrument stability and reproducibility during LC-MS analysis.”….. Please report QC metrics (e.g., PCA of QC clustering, RSD cutoff and % features removed, TIC stability, and features retained) in the main text or Supplement. If no RSD filter was applied, justify. These details are needed to support data quality claims.

**Response:**

We appreciate the reviewer’s insightful suggestion. Pooled QC samples were injected after every ten study samples. QC assessment on raw intensities showed that 3 826 of 3 913 features (97.8 %) passed the RSD cutoff of 30 %, indicating high reproducibility. RSD values ranged from 1.0 % to 105.1 %, with a median of 9.8 % and mean of 12.1 %. Principal component analysis (PCA) revealed tight clustering of QC injections. Total ion current (TIC) was calculated for all QC injections to monitor overall signal stability. TIC values were highly consistent, with a mean of 9.02 × 10¹⁰ and a standard deviation of 1.93 × 10⁹, resulting in a coefficient of variation (CV) of 2.14 %. The TIC plot showed no systematic drift across injections, confirming excellent instrument performance and reproducibility throughout the analytical run.

No RSD-based filtering was applied because QC evaluation showed that the majority of features were highly reproducible. Median RSD across QC injections was 9.8 %, 75 % of features had RSD < 15 %, and TIC CV was 2.14 %, indicating good instrument stability. Moreover, retaining all detected features minimizes potential bias and preserves low-abundance but biologically relevant metabolites that might otherwise be excluded by strict RSD thresholds. Therefore, all 3,913 detected features were retained for downstream analysis. Furthermore, all relevant details have been incorporated into the manuscript and supplementary figures.

Lines 163-164: “P-values were adjusted using the Benjamini-Hochberg correction.”…. The Methods state that p-values were adjusted using Benjamini–Hochberg, but the Results/Abstract report significance thresholds (p<0.05) without making clear whether quoted counts reflect adjusted q-values. Please revise all counts in the Abstract and Results so they explicitly say whether they are raw p or FDR q counts.

**Response:**

We thank the reviewer for the comment. To ensure clarity, all counts of significant metabolites in the Abstract and Results have been revised to explicitly indicate that they reflect **q-values** rather than raw p-values.

Results

Lines 170-171 and 188-190: “Untargeted metabolomic profiling identified 3,913 metabolites in back fat tissues of Ningxiang pigs across four developmental stages (180, 240, 300, and 360 days).” And “To identify temporally regulated metabolites and assess their functional properties, we next performed differential abundance analysis and identified 154 metabolites that significantly varied across the four developmental stages.”…………… Please distinguish between detected features (3,913) and annotated metabolites. For the 154 significant ones, indicate how many are high-confidence (MSI 1–2) vs. putative (MSI 3–4).

**Response:**

To clarify, the 3,913 features reported represent **all detected metabolic features** from untargeted profiling, whereas the 154 significant metabolites refer to **annotated metabolites** that showed temporal variation across developmental stages. To make it more easily distinguishable, we have revised the sentence, the relevant sentence reads: “**Among the 3,913 detected features, we performed differential abundance analysis to identify temporally regulated metabolites and assess their functional properties. This analysis revealed 154 metabolites that significantly varied across the four developmental stages**”.

We have indicated that 63 metabolites were MSI 1-2, The relevant sentence reads: “**Of these, 63 were annotated with KEGG and HMDB databases (MSI 1–2), while the remaining metabolites were putatively annotated (MSI 3–4)”**.

Lines 144-145 and 246-248: “WGCNA identified 37 different modules, each assigned with a distinct color, with highly correlated metabolites grouped together.” And “Thirty-six modules were 12 constructed (Fig 6B) and further used to build up module-trait relationships along with our previous published carcass traits [29] (Fig 7).”………..The manuscript inconsistently reports 36 vs. 37 modules. Please make this consistent, clarify whether the grey module was counted, and provide WGCNA parameters, module sizes, and the module–trait correlation matrix in the supplement.

**Response:**

Yes, the WGCNA analysis identified **37 modules in total, including the grey module**, which contains metabolites not assigned to any specific module. We have revised the text to consistently report 37 modules throughout the manuscript. Additionally, the **WGCNA parameters (soft-threshold power, TOM type, minimum module size, and merge cut height), module sizes, and the** **complete module–trait correlation matrix** have been provided in the Supplementary Materials (File S2-4)

Lines 146-147: “Our previously published carcass traits data were used to build up module-trait relationships [29].”….. Please clarify if carcass traits were measured on the same animals as the metabolomics samples. If yes, provide a table (IDs, trait values, normalization). If not, justify the use of external data and discuss the limitations of module–trait correlations.

**Response:**

Yes, the carcass traits were measured on the same Ningxiang pigs used for metabolomic profiling. A detailed table with individual animal IDs and trait values is provided in the File S4, However, our goal is goal is to correlate metabolites with **real, raw trait values** (e.g., body weight in kg), so it’s **better not to normalize** the traits.

Lines 208-214: “We also found that twenty-one metabolites were synthetic metabolites, with twelve of them accumulated from 160d to 360d, five increased at various stage, four gradually decreased in the back fat from 160d to 360d (Fig 5). In addition, we identified two mycotoxins, hydrolyzed fumonisin B1 (HFB1) and paspaline in the back fat. HFB1 was higher in the back fat at 160d and 360d, but paspaline was decreased from 240d (S1 File). The function of remaining metabolites was unknown due to lack of information in the database. Detailed information was listed in S1 File”……The Results section refers to 160d, but Abstract and Methods specify 180d as the earliest time point. Please correct these inconsistencies and ensure all text, figures, and supplements consistently use 180d, 240d, 300d, and 360d.

**Response:**

We have carefully checked the manuscript and corrected all inconsistencies. The earliest developmental stage is now consistently reported as **180 d** throughout the Abstract, Methods, Results, Figures, and Supplementary materials, with the four stages being **180d, 240d, 300d, and 360d**.

Reference

All references should be reformatted according to the journal’s reference style. Please ensure consistency in author listing, journal names, title capitalization, punctuation, and DOI formatting.

**Response:**

We have carefully reformatted all references to comply with the journal’s style, ensuring consistency in **author listing, journal names, title capitalization, punctuation, and DOI formatting**.
