# Supplementary material for: Metabolomic profiling of backfat in Ningxiang pigs reveals lipid dynamics and carcass trait associations during the fattening stage
Source: PLoS One. 2026 Jul 22;21(7):e0353743. doi: 10.1371/journal.pone.0353743 (PMC13390868; doi:10.1371/journal.pone.0353743)
Supplement: Attachment — Submitted filename: PONE-D-25-26633_R3 comments.docx [file pone.0353743.s017.docx]

The overall quality of the manuscript is good and the raised issues have been addressed properly; however, several minor language and wording issues should be corrected to improve readability. The specific suggestions are listed below.

**Line 110:** “Metabolites eluted from the column was detected with a high-resolution tandem mass spectrometer” replace with “Metabolites eluted from the column were detected with a high-resolution tandem mass spectrometer”

**Line 214:** Replace “methanandamide phosphate was deceased at 240d” with “methanandamide phosphate was decreased at 240d”

**Line 221:** Rephrase the sentence “Twelve were bioactive metabolites, including signaling alkaloids and antioxidants, were also temporally regulated” to “**Twelve bioactive metabolites**, including signaling alkaloids and antioxidants, were also temporally regulated.”

**Line 266:** “a cut-off of R² = 0.9” rewrite as “A cut-off of R² = 0.9”

**Line 441:** “While direct evidence linking pseudouridine to energy homeostasis is limited, but its synthesize enzyme, pseudouridine synthase,”…. It is “its synthesizing enzyme”.
