# Supplementary material for: Metabolomic profiling of backfat in Ningxiang pigs reveals lipid dynamics and carcass trait associations during the fattening stage
Source: PLoS One. 2026 Jul 22;21(7):e0353743. doi: 10.1371/journal.pone.0353743 (PMC13390868; doi:10.1371/journal.pone.0353743)
Supplement: Attachment — Submitted filename: Response to reviewers Mar 31.docx [file pone.0353743.s019.docx]

Reviewer #1:

#1 Should the term "back fat" in the full text be corrected to "backfat"?

We thank the reviewer for this suggestion. We have revised the manuscript by correcting all instances of 'back fat' to 'backfat', including those in the figures.

#2 Carcass traits do not include indicators such as organ weight and length.

These organ weight and length data (heart weight, lung weight, intestine length etc..) were already included in our experiment, as shown in Figure 7 and File S4.

#3 Is it necessary to supplement the genetic background of 24 pigs?

We have supplemented the genetic background information in the revised manuscript. It now reads “The 24 Ningxiang pigs at four developmental stages (180, 240, 300, and 360 days) were provided by Hunan Liushahe Ecological Animal Husbandry Co., Ltd., and all originated from the same closed breeding population to ensure genetic consistency.”

#4 Line 137: The DOI address is displayed as "Not found". Please verify and make necessary modifications.

We apologize for this. We have corrected the DOI.

#5 It is recommended to place data such as carcass traits in the appendix.

All raw carcass trait data were already included in our File S4.

Reviewer #2: I have reviewed the revised version of this manuscript. The authors have conscientiously addressed the previous comments and made substantial improvements to the paper. The revised manuscript shows significant enhancements in experimental validation and language quality. Therefore, I recommend this manuscript for acceptance.

We sincerely thank the reviewer for the positive feedback and for acknowledging the improvements made in the revised manuscript. We appreciate the reviewer’s time and expertise in evaluating our work.

Reviewer #3: The overall quality of the manuscript is good and the raised issues have been addressed properly; however, several minor language and wording issues should be corrected to improve readability. The specific suggestions are listed below.

We thank the reviewer for the positive assessment and for providing specific language and wording suggestions. We have carefully incorporated all of these corrections to enhance the manuscript's readability. Our point-by-point responses are as follows:

Line 110: “Metabolites eluted from the column was detected with a high-resolution tandem mass spectrometer” replace with “Metabolites eluted from the column were detected with a high-resolution tandem mass spectrometer”

We have corrected this grammatical error as suggested. The sentence now reads: “Metabolites eluted from the column were detected with a high-resolution tandem mass spectrometer.”

Line 214: Replace “methanandamide phosphate was deceased at 240d” with “methanandamide phosphate was decreased at 240d”

We thank the reviewer for catching this typo. We have replaced "deceased" with "decreased" in the revised manuscript.

Line 221: Rephrase the sentence “Twelve were bioactive metabolites, including signaling alkaloids and antioxidants, were also temporally regulated” to “Twelve bioactive metabolites, including signaling alkaloids and antioxidants, were also temporally regulated.”

We have rephrased the sentence as recommended. The corrected sentence now reads: “Twelve bioactive metabolites, including signaling alkaloids and antioxidants, were also temporally regulated.”

Line 266: “a cut-off of R² = 0.9” rewrite as “A cut-off of R² = 0.9”

We have revised this sentence to begin with a capital letter, as suggested.

Line 441: “While direct evidence linking pseudouridine to energy homeostasis is limited, but its synthesize enzyme, pseudouridine synthase,”…. It is “its synthesizing enzyme”.

We have corrected this sentence. The revised text now reads: “While direct evidence linking pseudouridine to energy homeostasis is limited, its synthesizing enzyme, pseudouridine synthase, …”
