# Supplementary material for: Metabolomic profiling of backfat in Ningxiang pigs reveals lipid dynamics and carcass trait associations during the fattening stage
Source: PLoS One. 2026 Jul 22;21(7):e0353743. doi: 10.1371/journal.pone.0353743 (PMC13390868; doi:10.1371/journal.pone.0353743)
Supplement: Attachment — Submitted filename: Response to reviewers May 19.docx [file pone.0353743.s020.docx]

Dear Editor,

Thank you very much for your careful evaluation of our revised manuscript and for your constructive comments. We sincerely appreciate your suggestions regarding the scientific rigor, language quality, and overall presentation of the manuscript.

In response to your comments, we have thoroughly revised the entire manuscript, including the abstract, to improve the scientific tone, grammar, and sentence structure. Informal expressions have been removed or rewritten to ensure a clearer, more formal, and academically appropriate style throughout the manuscript.

Furthermore, we have updated the reference list by incorporating several recent and relevant studies published in 2024–2025 to strengthen the scientific background and discussion of our work.

Thank you again for your valuable comments and consideration.
