# Supplementary material for: Metabolomic profiling of backfat in Ningxiang pigs reveals lipid dynamics and carcass trait associations during the fattening stage
Source: PLoS One. 2026 Jul 22;21(7):e0353743. doi: 10.1371/journal.pone.0353743 (PMC13390868; doi:10.1371/journal.pone.0353743)
Supplement: Peer review history

# Editor report: Decision Letter 0

**DOI:** 10.1371/journal.pone.0353743.r001

**Authors:** Aziz ur Rahman Muhammad

26 May 2025

PONE-D-25-26633

Metabolomic profiling of back fat in Ningxiang pigs: insights into lipid metabolism and carcass trait associations across later developmental stages

PLOS ONE

Dear Dr. Chen,

Thank you for submitting your manuscript to PLOS ONE. After careful consideration, we feel that it has merit but does not fully meet PLOS ONE’s publication criteria as it currently stands. Therefore, we invite you to submit a revised version of the manuscript that addresses the points raised during the review process.

Dear Authors

We have found multiple issue in your manuscirpt. I advised you to revise the manuscirpt before the review process. I have found grammatical inconsistencies multiple times in abstract section for example "Ningxiang pigs is a..." that should be "Ningxiang pigs are a...". Your abstract is overpacked and you have provided too much technical detail for an abstract, making it harder for readers to quickly grasp the study's significance. There is also lack of specificity in outcome interpretation for example the role of the bioactive and synthetic compounds isn't clearly contextualized. I would suggest you to simplify sentences and clarify the findings for example “Twelve DAMs were classified as bioactive compounds…”that should be “Twelve bioactive compounds were significantly altered, with three increasing and nine decreasing across developmental stages.”

In introduction section, I have found redundancy in your text. Several statements repeat ideas (e.g., flavor and fatty acid uniqueness are discussed in multiple ways) multiple time. You introduction section lack hypothesis for example the rationale behind selecting specific developmental stages isn't clearly stated in introduction section. I would like to suggest you to revise the introduction section and clearly state the hypothesis at the end of the introduction.

“We hypothesize that distinct metabolic profiles in back fat develop over time and correlate with key carcass traits.”

In M and M section, I have found that there is insufficient detail in WGCNA setup: Module merging criteria and method validation are not discussed. Furthermore, ethical statement buried in a paragraph: Hard to locate. Please add a brief validation comment on the WGCNA module significance (e.g., permutation testing) and format the ethical statement as a standalone subsection.

In result section, you overly descriptive with little interpretation: For example, “Fig 1 shows PC1 and PC2...” is descriptive without biological insight you used inconsistent terminology several time for example Sometimes “DAMs,” other times “metabolites” or “chemicals.” I suggest you to add brief biological implications under each figure for example “These lipids may contribute to the increased fat deposition observed at later stages.” And don’t forget to use consistent terminology (DAMs/metabolites).

In discussion section, similar information appears in the introduction and discussion. And authors failed to fully explore the results in this section. Authors provided limited integration of findings and discussion does not fully connect WGCNA and metabolite trends to pig physiology. I suggest you to synthesize findings instead of listing them for example, “The increased abundance of oleoylethanolamide at 300d suggests a possible regulatory shift toward reduced feed intake, potentially explaining the observed feed efficiency at later stages.”

Conclusion is too vague to understand and the conclusion doesn’t strongly restate the importance or novelty of the work.

I have found grammar and syntax errors throughout the manuscript. Authors used inconsistent use of past and present tense. I suggest authors for thorough proofreading by a native English speaker or professional editing service. Please maintain past tense for describing methods and results, present tense for general knowledge.

Please submit your revised manuscript by Jul 10 2025 11:59PM. If you will need more time than this to complete your revisions, please reply to this message or contact the journal office at plosone@plos.org. When you're ready to submit your revision, log on to https://www.editorialmanager.com/pone/ and select the 'Submissions Needing Revision' folder to locate your manuscript file.

Please include the following items when submitting your revised manuscript:

- A rebuttal letter that responds to each point raised by the academic editor and reviewer(s). You should upload this letter as a separate file labeled 'Response to Reviewers'.
- A marked-up copy of your manuscript that highlights changes made to the original version. You should upload this as a separate file labeled 'Revised Manuscript with Track Changes'.
- An unmarked version of your revised paper without tracked changes. You should upload this as a separate file labeled 'Manuscript'.

If you would like to make changes to your financial disclosure, please include your updated statement in your cover letter. Guidelines for resubmitting your figure files are available below the reviewer comments at the end of this letter.

If applicable, we recommend that you deposit your laboratory protocols in protocols.io to enhance the reproducibility of your results. Protocols.io assigns your protocol its own identifier (DOI) so that it can be cited independently in the future. For instructions see: https://journals.plos.org/plosone/s/submission-guidelines#loc-laboratory-protocols. Additionally, PLOS ONE offers an option for publishing peer-reviewed Lab Protocol articles, which describe protocols hosted on protocols.io. Read more information on sharing protocols at https://plos.org/protocols?utm_medium=editorial-email&utm_source=authorletters&utm_campaign=protocols.

We look forward to receiving your revised manuscript.

Kind regards,

Aziz ur Rahman Muhammad

Academic Editor

PLOS ONE

Journal requirements:

When submitting your revision, we need you to address these additional requirements.

1. Please ensure that your manuscript meets PLOS ONE's style requirements, including those for file naming. The PLOS ONE style templates can be found at

https://journals.plos.org/plosone/s/file?id=wjVg/PLOSOne_formatting_sample_main_body.pdf and

https://journals.plos.org/plosone/s/file?id=ba62/PLOSOne_formatting_sample_title_authors_affiliations.pdf

2. To comply with PLOS ONE submissions requirements, in your Methods section, please provide additional information regarding the experiments involving animals and ensure you have included details on (1) methods of sacrifice, (2) methods of anesthesia and/or analgesia, and (3) efforts to alleviate suffering.

3. Please note that PLOS ONE has specific guidelines on code sharing for submissions in which author-generated code underpins the findings in the manuscript. In these cases, we expect all author-generated code to be made available without restrictions upon publication of the work. Please review our guidelines at https://journals.plos.org/plosone/s/materials-and-software-sharing#loc-sharing-code and ensure that your code is shared in a way that follows best practice and facilitates reproducibility and reuse.

4. Thank you for uploading your study's underlying data set. Unfortunately, the repository you have noted in your Data Availability statement does not qualify as an acceptable data repository according to PLOS's standards.

At this time, please upload the minimal data set necessary to replicate your study's findings to a stable, public repository (such as figshare or Dryad) and provide us with the relevant URLs, DOIs, or accession numbers that may be used to access these data. For a list of recommended repositories and additional information on PLOS standards for data deposition, please see https://journals.plos.org/plosone/s/recommended-repositories.

5. We note that Figure 9 in your submission contain copyrighted images. All PLOS content is published under the Creative Commons Attribution License (CC BY 4.0), which means that the manuscript, images, and Supporting Information files will be freely available online, and any third party is permitted to access, download, copy, distribute, and use these materials in any way, even commercially, with proper attribution. For more information, see our copyright guidelines: http://journals.plos.org/plosone/s/licenses-and-copyright.

We require you to either (1) present written permission from the copyright holder to publish these figures specifically under the CC BY 4.0 license, or (2) remove the figures from your submission:

1. You may seek permission from the original copyright holder of Figure 9 to publish the content specifically under the CC BY 4.0 license.

We recommend that you contact the original copyright holder with the Content Permission Form (http://journals.plos.org/plosone/s/file?id=7c09/content-permission-form.pdf) and the following text:

“I request permission for the open-access journal PLOS ONE to publish XXX under the Creative Commons Attribution License (CCAL) CC BY 4.0 (http://creativecommons.org/licenses/by/4.0/). Please be aware that this license allows unrestricted use and distribution, even commercially, by third parties. Please reply and provide explicit written permission to publish XXX under a CC BY license and complete the attached form.”

Please upload the completed Content Permission Form or other proof of granted permissions as an "Other" file with your submission.

In the figure caption of the copyrighted figure, please include the following text: “Reprinted from [ref] under a CC BY license, with permission from [name of publisher], original copyright [original copyright year].”

2. If you are unable to obtain permission from the original copyright holder to publish these figures under the CC BY 4.0 license or if the copyright holder’s requirements are incompatible with the CC BY 4.0 license, please either i) remove the figure or ii) supply a replacement figure that complies with the CC BY 4.0 license. Please check copyright information on all replacement figures and update the figure caption with source information. If applicable, please specify in the figure caption text when a figure is similar but not identical to the original image and is therefore for illustrative purposes only.

6. We note that there is identifying data in the Supporting Information file <Supplementary Material S1.xlsx>. Due to the inclusion of these potentially identifying data, we have removed this file from your file inventory. Prior to sharing human research participant data, authors should consult with an ethics committee to ensure data are shared in accordance with participant consent and all applicable local laws.

Data sharing should never compromise participant privacy. It is therefore not appropriate to publicly share personally identifiable data on human research participants. The following are examples of data that should not be shared:

-Name, initials, physical address

-Ages more specific than whole numbers

-Internet protocol (IP) address

-Specific dates (birth dates, death dates, examination dates, etc.)

-Contact information such as phone number or email address

-Location data

-ID numbers that seem specific (long numbers, include initials, titled “Hospital ID”) rather than random (small numbers in numerical order)

Data that are not directly identifying may also be inappropriate to share, as in combination they can become identifying. For example, data collected from a small group of participants, vulnerable populations, or private groups should not be shared if they involve indirect identifiers (such as sex, ethnicity, location, etc.) that may risk the identification of study participants.

Additional guidance on preparing raw data for publication can be found in our Data Policy (https://journals.plos.org/plosone/s/data-availability#loc-human-research-participant-data-and-other-sensitive-data) and in the following article: http://www.bmj.com/content/340/bmj.c181.long.

Please remove or anonymize all personal information, ensure that the data shared are in accordance with participant consent, and re-upload a fully anonymized data set. Please note that spreadsheet columns with personal information must be removed and not hidden as all hidden columns will appear in the published file.

Additional Editor Comments:

Dear Authors

I have found multiple issue in your manuscirpt. I advised you to revise the manuscirpt before the review process. I have found grammatical inconsistencies multiple times in abstract section for example "Ningxiang pigs is a..." that should be "Ningxiang pigs are a...". Your abstract is overpacked and you have provided too much technical detail for an abstract, making it harder for readers to quickly grasp the study's significance. There is also lack of specificity in outcome interpretation for example the role of the bioactive and synthetic compounds isn't clearly contextualized. I would suggest you to simplify sentences and clarify the findings for example “Twelve DAMs were classified as bioactive compounds…”that should be “Twelve bioactive compounds were significantly altered, with three increasing and nine decreasing across developmental stages.”

In introduction section, I have found redundancy in your text. Several statements repeat ideas (e.g., flavor and fatty acid uniqueness are discussed in multiple ways) multiple time. You introduction section lack hypothesis for example the rationale behind selecting specific developmental stages isn't clearly stated in introduction section. I would like to suggest you to revise the introduction section and clearly state the hypothesis at the end of the introduction.

“We hypothesize that distinct metabolic profiles in back fat develop over time and correlate with key carcass traits.”

In M and M section, I have found that there is insufficient detail in WGCNA setup: Module merging criteria and method validation are not discussed. Furthermore, ethical statement buried in a paragraph: Hard to locate. Please add a brief validation comment on the WGCNA module significance (e.g., permutation testing) and format the ethical statement as a standalone subsection.

In result section, you overly descriptive with little interpretation: For example, “Fig 1 shows PC1 and PC2...” is descriptive without biological insight you used inconsistent terminology several time for example Sometimes “DAMs,” other times “metabolites” or “chemicals.” I suggest you to add brief biological implications under each figure for example “These lipids may contribute to the increased fat deposition observed at later stages.” And don’t forget to use consistent terminology (DAMs/metabolites).

In discussion section, similar information appears in the introduction and discussion. And authors failed to fully explore the results in this section. Authors provided limited integration of findings and discussion does not fully connect WGCNA and metabolite trends to pig physiology. I suggest you to synthesize findings instead of listing them for example, “The increased abundance of oleoylethanolamide at 300d suggests a possible regulatory shift toward reduced feed intake, potentially explaining the observed feed efficiency at later stages.”

Conclusion is too vague to understand and the conclusion doesn’t strongly restate the importance or novelty of the work.

I have found grammar and syntax errors throughout the manuscript. Authors used inconsistent use of past and present tense. I suggest authors for thorough proofreading by a native English speaker or professional editing service. Please maintain past tense for describing methods and results, present tense for general knowledge.

[Note: HTML markup is below. Please do not edit.]

[NOTE: If reviewer comments were submitted as an attachment file, they will be attached to this email and accessible via the submission site. Please log into your account, locate the manuscript record, and check for the action link "View Attachments". If this link does not appear, there are no attachment files.]

While revising your submission, please upload your figure files to the Preflight Analysis and Conversion Engine (PACE) digital diagnostic tool, https://pacev2.apexcovantage.com/. PACE helps ensure that figures meet PLOS requirements. To use PACE, you must first register as a user. Registration is free. Then, login and navigate to the UPLOAD tab, where you will find detailed instructions on how to use the tool. If you encounter any issues or have any questions when using PACE, please email PLOS at figures@plos.org. Please note that Supporting Information files do not need this step.

# Author comment: Author response to Decision Letter 1

**DOI:** 10.1371/journal.pone.0353743.r002

25 Jun 2025

Thank you for your valuable comments and suggestions. We sincerely appreciate your time and effort in reviewing our manuscript. We have carefully revised the manuscript and addressed each of the issues point by point below.

I have found grammatical inconsistencies multiple times in abstract section for example "Ningxiang pigs is a..." that should be "Ningxiang pigs are a...".

Your abstract is overpacked and you have provided too much technical detail for an abstract, making it harder for readers to quickly grasp the study's significance.

There is also lack of specificity in outcome interpretation for example the role of the bioactive and synthetic compounds isn't clearly contextualized.

I would suggest you to simplify sentences and clarify the findings for example “Twelve DAMs were classified as bioactive compounds…”that should be “Twelve bioactive compounds were significantly altered, with three increasing and nine decreasing across developmental stages.”

We have corrected all grammatical errors, including the specific example mentioned. The abstract has been substantially streamlined to focus on the most essential information. We have clarified the biological and practical significance of the key findings, particularly regarding the roles of bioactive and synthetic metabolites. Also, we have revised sentences throughout the entire abstract to improve flow and precision.

In introduction section, I have found redundancy in your text. Several statements repeat ideas (e.g., flavor and fatty acid uniqueness are discussed in multiple ways) multiple time.

You introduction section lack hypothesis, for example the rationale behind selecting specific developmental stages isn't clearly stated in introduction section. I would like to suggest you to revise the introduction section and clearly state the hypothesis at the end of the introduction.

“We hypothesize that distinct metabolic profiles in back fat develop over time and correlate with key carcass traits.”

We have carefully revised the Introduction to eliminate repetitive statements, particularly those related to fat flavor and fatty acid composition. We agree that a clear hypothesis strengthens the focus of the study. As suggested, we have now explicitly stated the rationale for selecting the fattening stage (180–360 days) and included the hypothesis at the end of the Introduction. The revised statement reads: “We hypothesize that developmental stage-specific changes in the back fat metabolome of Ningxiang pigs reflect coordinated regulation of energy metabolism, and key metabolites within this profile are associated with economically important carcass traits and breeding performance.”

In M and M section, I have found that there is insufficient detail in WGCNA setup: Module merging criteria and method validation are not discussed.

Furthermore, ethical statement buried in a paragraph: Hard to locate. Please add a brief validation comment on the WGCNA module significance (e.g., permutation testing) and format the ethical statement as a standalone subsection.

We have added the detail in WGCNA setup to corresponding locations in methods:

“Modules were merged based on eigengene similarity using a cut height of 0.25 (correlation > 0.75). The biological relevance of modules was validated by correlating eigengenes with developmental stages. “

“Permutation testing (n = 1000) confirmed that significant module–trait associations were unlikely to occur by chance (p<0.05), supporting the robustness of the identified modules.”

We agree that the ethical approval details were not prominently displayed. We have now reformatted the ethical statement into a clearly labeled, standalone subsection titled “Animal ethics” to improve readability and compliance with standard reporting practices.

In result section, you overly descriptive with little interpretation: For example, “Fig 1 shows PC1 and PC2...” is descriptive without biological insight

you used inconsistent terminology several time for example Sometimes “DAMs,” other times “metabolites” or “chemicals.” I suggest you to add brief biological implications under each figure for example “These lipids may contribute to the increased fat deposition observed at later stages.” And don’t forget to use consistent terminology (DAMs/metabolites).

We agree that parts of the Results section were overly descriptive and lacked sufficient biological interpretation. In response, we have added the relevant sections to include more insight into the biological significance of the findings. We have replaced “DAMs” and chemicals with metabolites to keep the consistency. In addition, we have added brief biological implications under each figure.

In discussion section, similar information appears in the introduction and discussion. And authors failed to fully explore the results in this section. Authors provided limited integration of findings and discussion does not fully connect WGCNA and metabolite trends to pig physiology. I suggest you to synthesize findings instead of listing them for example, “The increased abundance of oleoylethanolamide at 300d suggests a possible regulatory shift toward reduced feed intake, potentially explaining the observed feed efficiency at later stages.”

We have removed repetitive background information from the discussion that was already covered in the introduction. We restructured the discussion to better integrate and interpret the metabolite trends in the context of pig physiology. Rather than listing individual metabolite changes, we now emphasize overarching patterns, such as temporal shifts in lipid and bioactive compounds, and their biological implications, including appetite regulation, oxidative stress, etc. Additionally, we strengthened the connection between WGCNA modules and carcass traits by contextualizing hub metabolites (e.g., psychotrine, uridine, pseudouridine) with known physiological functions such as energy metabolism and mitochondrial regulation.

Conclusion is too vague to understand and the conclusion doesn’t strongly restate the importance or novelty of the work.

We have rewritten the conclusion to be more specific and impactful.

I have found grammar and syntax errors throughout the manuscript. Authors used inconsistent use of past and present tense. I suggest authors for thorough proofreading by a native English speaker or professional editing service. Please maintain past tense for describing methods and results, present tense for general knowledge.

We have used a professional editing tool to correct grammatical errors throughout the entire manuscript. In addition, we would like to clarify that, in accordance with standard scientific writing conventions, we have used the past tense when describing our experimental design in the Introduction and when discussing our findings in the Discussion section.

Journal requirements:

When submitting your revision, we need you to address these additional requirements.

1. Please ensure that your manuscript meets PLOS ONE's style requirements, including those for file naming. The PLOS ONE style templates can be found at

https://journals.plos.org/plosone/s/file?id=wjVg/PLOSOne_formatting_sample_main_body.pdf and

https://journals.plos.org/plosone/s/file?id=ba62/PLOSOne_formatting_sample_title_authors_affiliations.pdf

We have ensured that our manuscript meets the journal style requirements.

2. To comply with PLOS ONE submissions requirements, in your Methods section, please provide additional information regarding the experiments involving animals and ensure you have included details on (1) methods of sacrifice, (2) methods of anesthesia and/or analgesia, and (3) efforts to alleviate suffering.

We have included additional information regarding the animal handling and ethics in methods section: “The pigs were provided with free access to clean water and a nutritionally balanced diet throughout the study. They were monitored daily for signs of illness or distress, and any sick or injured animals received prompt veterinary care in accordance with institutional guidelines. The pigs were humanely euthanized using electrical stunning followed by slaughter.”

3. Please note that PLOS ONE has specific guidelines on code sharing for submissions in which author-generated code underpins the findings in the manuscript. In these cases, we expect all author-generated code to be made available without restrictions upon publication of the work. Please review our guidelines at https://journals.plos.org/plosone/s/materials-and-software-sharing#loc-sharing-code and ensure that your code is shared in a way that follows best practice and facilitates reproducibility and reuse.

We have uploaded our R code on protocols.io, http://dx.doi.org/10.17504/protocols.io.kqdg3wj51v25/v1, and added the doi link to the method section.

4. Thank you for uploading your study's underlying data set. Unfortunately, the repository you have noted in your Data Availability statement does not qualify as an acceptable data repository according to PLOS's standards.

At this time, please upload the minimal data set necessary to replicate your study's findings to a stable, public repository (such as figshare or Dryad) and provide us with the relevant URLs, DOIs, or accession numbers that may be used to access these data. For a list of recommended repositories and additional information on PLOS standards for data deposition, please see https://journals.plos.org/plosone/s/recommended-repositories.

We have revised Data Availability statement.

The raw data will be available upon publication: Figshare: 10.6084/m9.figshare.29361956.

For reviewers to access, please use private link: https://figshare.com/s/2c28cc686518afd040b2

5. We note that Figure 9 in your submission contain copyrighted images. All PLOS content is published under the Creative Commons Attribution License (CC BY 4.0), which means that the manuscript, images, and Supporting Information files will be freely available online, and any third party is permitted to access, download, copy, distribute, and use these materials in any way, even commercially, with proper attribution. For more information, see our copyright guidelines: http://journals.plos.org/plosone/s/licenses-and-copyright.

We require you to either (1) present written permission from the copyright holder to publish these figures specifically under the CC BY 4.0 license, or (2) remove the figures from your submission:

We have replaced the image in Fig 9 with a new version that does not have any copyright issues.

6. We note that there is identifying data in the Supporting Information file <Supplementary Material S1.xlsx>. Due to the inclusion of these potentially identifying data, we have removed this file from your file inventory. Prior to sharing human research participant data, authors should consult with an ethics committee to ensure data are shared in accordance with participant consent and all applicable local laws.

We would like to clarify that our study did not involve human research participants. The data provided in Supplementary Material S1.xlsx are exclusively derived from pigs used in the animal experiment.

We have reviewed the spreadsheet and can confirm that it does not contain any personally identifiable information or sensitive data related to humans. All identifiers in the file pertain solely to animals and are used for tracking purposes within the study (e.g., animal stage), which do not pose any privacy or ethical concerns.

We will upload the file again if it is needed.

Attachment Submitted filename: Response to Reviewers.docx

# Editor report: Decision Letter 1

**DOI:** 10.1371/journal.pone.0353743.r003

**Authors:** Aziz ur Rahman Muhammad, Aziz ur Rahman Muhammad

21 Jul 2025

PONE-D-25-26633R1

Metabolomic profiling of back fat in Ningxiang pigs reveals lipid dynamics and carcass trait associations during the fattening stage

PLOS ONE

Dear Dr. Chen,

Thank you for submitting your manuscript to PLOS ONE. After careful consideration, we feel that it has merit but does not fully meet PLOS ONE’s publication criteria as it currently stands. Therefore, we invite you to submit a revised version of the manuscript that addresses the points raised during the review process.

The revised manuscript has addressed several of the initial concerns, but significant issues remain that affect the overall quality and clarity of the work. The abstract is still overly dense and lacks proper structure. It should be concise and formatted as a single paragraph. Statistical descriptions (e.g., p-values, effect sizes) are missing, which are critical for interpreting the significance of the findings. Please also improver inroduciton, M and M section, Results and discussion section in revision and resubmit.

Please submit your revised manuscript by Sep 04 2025 11:59PM. If you will need more time than this to complete your revisions, please reply to this message or contact the journal office at plosone@plos.org. When you're ready to submit your revision, log on to https://www.editorialmanager.com/pone/ and select the 'Submissions Needing Revision' folder to locate your manuscript file.

Please include the following items when submitting your revised manuscript:

- A rebuttal letter that responds to each point raised by the academic editor and reviewer(s). You should upload this letter as a separate file labeled 'Response to Reviewers'.
- A marked-up copy of your manuscript that highlights changes made to the original version. You should upload this as a separate file labeled 'Revised Manuscript with Track Changes'.
- An unmarked version of your revised paper without tracked changes. You should upload this as a separate file labeled 'Manuscript'.

If you would like to make changes to your financial disclosure, please include your updated statement in your cover letter. Guidelines for resubmitting your figure files are available below the reviewer comments at the end of this letter.

If applicable, we recommend that you deposit your laboratory protocols in protocols.io to enhance the reproducibility of your results. Protocols.io assigns your protocol its own identifier (DOI) so that it can be cited independently in the future. For instructions see: https://journals.plos.org/plosone/s/submission-guidelines#loc-laboratory-protocols. Additionally, PLOS ONE offers an option for publishing peer-reviewed Lab Protocol articles, which describe protocols hosted on protocols.io. Read more information on sharing protocols at https://plos.org/protocols?utm_medium=editorial-email&utm_source=authorletters&utm_campaign=protocols.

We look forward to receiving your revised manuscript.

Kind regards,

Aziz ur Rahman Muhammad

Academic Editor

PLOS ONE

Journal Requirements:

1. If the reviewer comments include a recommendation to cite specific previously published works, please review and evaluate these publications to determine whether they are relevant and should be cited. There is no requirement to cite these works unless the editor has indicated otherwise.

Additional Editor Comments:

Dear Authors

The revised manuscript has addressed several of the initial concerns, but significant issues remain that affect the overall quality and clarity of the work. Below are the key gaps and problems that warrant rejection:

The abstract is still overly dense and lacks proper structure. It should be concise and formatted as a single paragraph. Statistical descriptions (e.g., p-values, effect sizes) are missing, which are critical for interpreting the significance of the findings. The abstract should clearly state the novelty and broader implications of the study, which are currently unclear.

I have also found inconsistent terminologies throughout the manuscript for example manuscript still uses terms like "DAMs" (differentially abundant metabolites) interchangeably with "metabolites" or "chemicals," which can confuse readers. Moreover, the results section remains overly descriptive without sufficient biological context. For example, the PCA analysis (Fig 1) is described but not linked to meaningful biological shifts in fat metabolism. The discussion of bioactive and synthetic compounds lacks depth. The implications for pig health or meat quality are not fully explored. Furthermore, statistical details (e.g., ANOVA results, post-hoc test outcomes) are either missing or buried in supplementary files. These should be highlighted in the main text. The WGCNA analysis lacks clarity in how modules were validated or how hub metabolites were selected.

The discussion fails to synthesize findings into a coherent narrative. For example, the role of psychotrine in carcass traits is mentioned but not contextualized within existing literature. The health risks of synthetic compounds are noted, but their practical implications for breeding or food safety are not discussed in depth. At the end I would say that conclusion is vague and does not strongly restate the study's novelty or actionable insights. It should explicitly highlight how the findings advance the field.

[Note: HTML markup is below. Please do not edit.]

[NOTE: If reviewer comments were submitted as an attachment file, they will be attached to this email and accessible via the submission site. Please log into your account, locate the manuscript record, and check for the action link "View Attachments". If this link does not appear, there are no attachment files.]

While revising your submission, please upload your figure files to the Preflight Analysis and Conversion Engine (PACE) digital diagnostic tool, https://pacev2.apexcovantage.com/. PACE helps ensure that figures meet PLOS requirements. To use PACE, you must first register as a user. Registration is free. Then, login and navigate to the UPLOAD tab, where you will find detailed instructions on how to use the tool. If you encounter any issues or have any questions when using PACE, please email PLOS at figures@plos.org. Please note that Supporting Information files do not need this step.

# Author comment: Author response to Decision Letter 2

**DOI:** 10.1371/journal.pone.0353743.r004

28 Jul 2025

Dear Authors

The revised manuscript has addressed several of the initial concerns, but significant issues remain that affect the overall quality and clarity of the work. Below are the key gaps and problems that warrant rejection:

The abstract is still overly dense and lacks proper structure. It should be concise and formatted as a single paragraph. Statistical descriptions (e.g., p-values, effect sizes) are missing, which are critical for interpreting the significance of the findings. The abstract should clearly state the novelty and broader implications of the study, which are currently unclear.

We have revised the abstract to ensure it is concise, clearly structured as a single paragraph, and free from unnecessary density. P-values have been added to highlight the significance of our key findings. We also clarified the novelty and broader implications of the study to better communicate its scientific contribution.

I have also found inconsistent terminologies throughout the manuscript for example manuscript still uses terms like "DAMs" (differentially abundant metabolites) interchangeably with "metabolites" or "chemicals," which can confuse readers.

We agree that using terms like "DAMs," "metabolites," and "chemicals" interchangeably may cause confusion for readers. In response, we have carefully reviewed the entire manuscript and removed all instances of “DAMs” and other inconsistent or unclear terminology. We now consistently use the term “metabolites” throughout the text and figure labels to maintain clarity and uniformity. These revisions have been applied in the main text and figure legends as appropriate.

Moreover, the results section remains overly descriptive without sufficient biological context. For example, the PCA analysis (Fig 1) is described but not linked to meaningful biological shifts in fat metabolism.

We have revised and linked PCA to meaningful biological shifts in our results: “The metabolite profile at 180d was clearly separated from those at later stages (240–360d), contributing most to the variation along PC1. This suggests a significant metabolic shift occurring after 180 days, likely reflecting the onset of the fattening phase, where energy storage and lipid biosynthesis become more prominent.” Please note that PCA itself does not directly show biological processes, but only suggest when major biological shifts might occur.

The main purpose of PCA in this study was to explore overall variation in the metabolomic data and assess whether samples clustered by developmental stage. PCA is exploratory, not confirmatory. If PCA shows clear stage-based separation, it's reasonable to then prioritize those comparisons, but this decision should also be backed by following analysis.

We have also revised the rest part of results with sufficient biological context.

Furthermore, statistical details (e.g., ANOVA results, post-hoc test outcomes) are either missing or buried in supplementary files. These should be highlighted in the main text.

The statistical details, including ANOVA results and post-hoc test outcomes, were described in the “Statistics” section of the Methods and provided in full in the Supplementary Files due to the large number of pairwise comparisons across all metabolites. Given the scale of the data, it is not practical to list every statistical outcome in the main text or a table. Instead, we followed standard practice by visualizing key results using heatmaps and scatterplots with Compact Letter Display (CLD) to indicate statistically significant differences, as shown in Figures 2–5.

To address your concern and better highlight the significance of our findings, we have now: Added p-values (e.g., p < 0.05) directly in the figure legends and main text where appropriate, and ensured that visual indicators of significance (e.g., CLDs) are clearly described and linked to the underlying statistics.

We hope these changes improve the clarity and accessibility of the statistical results.

The WGCNA analysis lacks clarity in how modules were validated or how hub metabolites were selected.

These details were already described in both the Methods and Results sections, and we also uploaded our R scripts for clarity (as requested): The R code can be accessed from http://dx.doi.org/10.17504/protocols.io.kqdg3wj51v25/v1.

To make sure that it has been sufficiently emphasized, we further revised the relevant text (in methods) to clarify the analytical steps:

Module validation: To ensure robustness, we performed 1,000 permutation tests, confirming that the observed module–trait associations were unlikely to occur by chance (p < 0.05). Only modules with strong correlations (|R| > 0.5, p < 0.05) were included in downstream analysis.

Hub metabolite selection: Within each trait-associated module, we ranked metabolites based on their module membership (MM) values. Metabolites with the highest MM values were considered "hub metabolites," as they represent highly connected nodes within the network. Among these, we further prioritized hub metabolites that also showed significant correlation with carcass traits (|R| > 0.5, p < 0.05), ensuring both topological and phenotypic relevance.

The discussion fails to synthesize findings into a coherent narrative. For example, the role of psychotrine in carcass traits is mentioned but not contextualized within existing literature.

Psychotrine is an underexplored metabolite, with only nine publications listed in PubMed and none addressing its role in animal growth or carcass traits. Our identification of psychotrine as the hub metabolite in a module strongly correlated with key carcass performance indicators—alongside decoquinate, a known growth-promoting agent—suggests a previously unrecognized link between this emetine-class alkaloid and livestock development. This co-occurrence within a growth-related metabolic network presents a novel finding that expands current understanding of carcass trait regulation and highlights psychotrine as a promising candidate for further functional investigation in swine physiology.

We have revised the discussion to emphasize the novelty of this finding, highlighting psychotrine as a previously uncharacterized metabolite potentially associated with growth-related traits in swine.

The discussion of bioactive and synthetic compounds lacks depth.

We have substantially revised the discussion to provide deeper insight into the biological relevance of both bioactive and synthetic compounds identified in back fat. Specifically, we expanded the interpretation of several key metabolites by discussing their known functional roles, sources, and potential physiological implications.

The health risks of synthetic compounds are noted, but their practical implications for breeding or food safety are not discussed in depth.

We have revised the discussion section to better articulate the practical implications of our findings.

The implications for pig health or meat quality are not fully explored.

In the revised discussion section, we have expanded on the implications of our findings for pig health and meat quality. Specifically, we discuss how shifts in metabolite profiles—such as increased antioxidant compounds (e.g., α-tocotrienol) and decreased pro-inflammatory or oxidative stress–related metabolites (e.g., pentosidine)—may indicate improved metabolic resilience and fat quality during the fattening period. Conversely, the accumulation of synthetic or potentially harmful exogenous compounds raises concerns regarding long-term animal health and pork safety. These findings offer valuable insights for optimizing feeding practices, improving meat quality traits, and informing breeding strategies aimed at enhancing metabolic efficiency and minimizing contaminant accumulation.

At the end I would say that conclusion is vague and does not strongly restate the study's novelty or actionable insights. It should explicitly highlight how the findings advance the field.

We have revised the conclusion to more clearly articulate the study's novelty and practical implications.

Attachment Submitted filename: Response_to_Reviewers_auresp_2.docx

# Peer review: Decision Letter 2

**DOI:** 10.1371/journal.pone.0353743.r005

**Authors:** Aziz ur Rahman Muhammad, Aziz ur Rahman Muhammad, Aziz ur Rahman Muhammad

8 Oct 2025

<div>PONE-D-25-26633R2

Metabolomic profiling of back fat in Ningxiang pigs reveals lipid dynamics and carcass trait associations during the fattening stage

PLOS ONE

Dear Dr. Chen,

Thank you for submitting your manuscript to PLOS ONE. After careful consideration, we feel that it has merit but does not fully meet PLOS ONE’s publication criteria as it currently stands. Therefore, we invite you to submit a revised version of the manuscript that addresses the points raised during the review process.

Please improve methodology transparency, data consistency, and interpretation. Issues include unclear QC validation, inconsistent timepoints and module reporting, and insufficient explanation of statistical corrections and metabolite annotation. Additionally, language, formatting, and figure labeling need improvement to ensure clarity, accuracy, and compliance with journal standards.

Please submit your revised manuscript by Nov 22 2025 11:59PM. If you will need more time than this to complete your revisions, please reply to this message or contact the journal office at plosone@plos.org. When you're ready to submit your revision, log on to https://www.editorialmanager.com/pone/ and select the 'Submissions Needing Revision' folder to locate your manuscript file.

Please include the following items when submitting your revised manuscript:

- A rebuttal letter that responds to each point raised by the academic editor and reviewer(s). You should upload this letter as a separate file labeled 'Response to Reviewers'.
- A marked-up copy of your manuscript that highlights changes made to the original version. You should upload this as a separate file labeled 'Revised Manuscript with Track Changes'.
- An unmarked version of your revised paper without tracked changes. You should upload this as a separate file labeled 'Manuscript'.

If you would like to make changes to your financial disclosure, please include your updated statement in your cover letter. Guidelines for resubmitting your figure files are available below the reviewer comments at the end of this letter.

If applicable, we recommend that you deposit your laboratory protocols in protocols.io to enhance the reproducibility of your results. Protocols.io assigns your protocol its own identifier (DOI) so that it can be cited independently in the future. For instructions see: https://journals.plos.org/plosone/s/submission-guidelines#loc-laboratory-protocols. Additionally, PLOS ONE offers an option for publishing peer-reviewed Lab Protocol articles, which describe protocols hosted on protocols.io. Read more information on sharing protocols at https://plos.org/protocols?utm_medium=editorial-email&utm_source=authorletters&utm_campaign=protocols.

We look forward to receiving your revised manuscript.

Kind regards,

Aziz ur Rahman Muhammad

Academic Editor

PLOS ONE

Journal Requirements:

If the reviewer comments include a recommendation to cite specific previously published works, please review and evaluate these publications to determine whether they are relevant and should be cited. There is no requirement to cite these works unless the editor has indicated otherwise.

Additional Editor Comments:

The study presents a valuable metabolomic analysis of backfat development in Ningxiang pigs, but requires major revisions in methodology transparency, data consistency, and interpretation. Issues include unclear QC validation, inconsistent timepoints and module reporting, and insufficient explanation of statistical corrections and metabolite annotation. Additionally, language, formatting, and figure labeling need improvement to ensure clarity, accuracy, and compliance with journal standards.

[Note: HTML markup is below. Please do not edit.]

Reviewers' comments:

Reviewer's Responses to Questions

**Comments to the Author**

1. If the authors have adequately addressed your comments raised in a previous round of review and you feel that this manuscript is now acceptable for publication, you may indicate that here to bypass the “Comments to the Author” section, enter your conflict of interest statement in the “Confidential to Editor” section, and submit your "Accept" recommendation.

Reviewer #1: (No Response)

Reviewer #2: (No Response)

Reviewer #3: (No Response)

**********

2. Is the manuscript technically sound, and do the data support the conclusions?

The manuscript must describe a technically sound piece of scientific research with data that supports the conclusions. Experiments must have been conducted rigorously, with appropriate controls, replication, and sample sizes. The conclusions must be drawn appropriately based on the data presented.

Reviewer #1: Yes

Reviewer #2: Partly

Reviewer #3: No

**********

3. Has the statistical analysis been performed appropriately and rigorously?

Reviewer #1: Yes

Reviewer #2: Yes

Reviewer #3: No

**********

4. Have the authors made all data underlying the findings in their manuscript fully available?

The PLOS Data policy requires authors to make all data underlying the findings described in their manuscript fully available without restriction, with rare exception (please refer to the Data Availability Statement in the manuscript PDF file). The data should be provided as part of the manuscript or its supporting information, or deposited to a public repository. For example, in addition to summary statistics, the data points behind means, medians and variance measures should be available. If there are restrictions on publicly sharing data—e.g. participant privacy or use of data from a third party—those must be specified.

Reviewer #1: Yes

Reviewer #2: Yes

Reviewer #3: Yes

**********

5. Is the manuscript presented in an intelligible fashion and written in standard English?

PLOS ONE does not copyedit accepted manuscripts, so the language in submitted articles must be clear, correct, and unambiguous. Any typographical or grammatical errors should be corrected at revision, so please note any specific errors here.

Reviewer #1: Yes

Reviewer #2: (No Response)

Reviewer #3: Yes

**********

6. Review Comments to the Author

Please use the space provided to explain your answers to the questions above. You may also include additional comments for the author, including concerns about dual publication, research ethics, or publication ethics. (Please upload your review as an attachment if it exceeds 20,000 characters)

Reviewer #1: (1) The typical slaughter age for Ningxiang pigs is 8 months. However, in the study, the pigs were raised until they reached 360 days old. Has this exceeded the standard slaughter age requirement?

(2) I think that the PCA plot only indicates that there are significant differences among the four stages, but it does not represent the conclusion described by authors.

(3) The result does not seem to cover all the classification results of non-targeted metabolites.

(4) If there are other references for using metabolites for co-expression analysis, please add them to the methods section.

Reviewer #2: This study presents a metabolomic analysis of back fat in Ningxiang pigs across the fattening stage (180–360 days), a critical developmental window for lipid deposition and carcass quality. The work provided a metabolic atlas of backfat dynamics during this period in an indigenous pig breed. However, several issues require revision to improve clarity, accuracy, and rigor.

In abstract

1. Abbreviations in the text should be defined at their first appearance. For example, in the abstract (line: 30), 'weighted gene co-expression network' should be followed by their abbreviations. Additionally, 'weighted gene co-expression network' is missing the word 'analysis' (should be 'weighted gene co-expression network analysis (WGCNA). Similar issues may exist elsewhere; please conduct a thorough review of the entire manuscript.

2. Line 22–23: "metabolomic profile" should be changed to the plural form "profiles" because the study involves multiple time points.

3. Line 24, the less-than sign (<) in (p<0.05) should be formatted with spaces around the operator, i.e., "(p < 0.05)", as shown on line 30. Please conduct a thorough check throughout the entire manuscript.

In the Keywords, please change Sus scrofa domesticus to Ningxiang pigs

In introduction

1. In line 49: The thickness of "backfat" is a critical indicator in pig breeding and meat production". The term "backfat" appears in two different forms throughout the manuscript: "backfat" and "back fat". Please standardize and use "backfat" consistently throughout the entire text.

Line 59: Ningxiang pigs, like many Chinese indigenous pig breeds, reach sexual maturity relatively early (120 day(d)), but full body maturity occurs later (300-360d). Please delete day ().

Line 77-78: The pigs were provided with free access to clean water and a nutritionally balanced diet

78 throughout the study. Please change "provided with" to "had ad libitum access to".

Lines 85-89: The manuscript should include a clear description of the slaughter method used. Specifically, please clarify whether electrical stunning or intravenous injection (e.g., pentobarbital) was employed for euthanasia.

In Materials and Methods

Line 86It is recommended to provide the general composition of the diet used in the study in a supplementary table.

1. Line 132: The package "ggplot" should be corrected to "ggplot2", as "ggplot" is outdated and the current standard is "ggplot2". Additionally, please provide version numbers for all software and R packages used, such as ggplot2 and WGCNA, to ensure reproducibility. In addition, the statement "Detailed ANOVA and Tukey’s HSD test results were listed in the S1 File" could be improved. It is recommended to format the supplementary materials as clearly labeled tables (e.g., Table S1, Table S2, Table S3) to enhance readability and facilitate reference.

2. In line 150: Please ensure consistent use of lowercase "p" for p value throughout the manuscript (e.g., p < 0.05).

In Results

1. In line 171: Untargeted metabolomic profiling identified 3,913 metabolites in back fat tissues of 171 Ningxiang pigs across four developmental stages (180, 240, 300, and 360 days). Please provide the total number of metabolites identified at each developmental stage, and indicate in the text which supplementary table (e.g., Table S1, S2, etc.) contains the detailed metabolite data.

2. Please include the percentage of variance explained by each principal component (e.g., PC1 and PC2) on the axes of the PCA plot in Fig 1

3. There is an incorrect sequence in the statistical description in Lines 126–131. One-way ANOVA should first be used to test whether there are significant differences among all groups (overall significance). This is followed by P-value adjustment (e.g., Benjamini-Hochberg correction). Only for metabolites showing significant results in the ANOVA (after correction) should post-hoc pairwise comparisons (e.g., Tukey’s HSD test) be performed to identify which specific group pairs differ significantly. Additionally, the sentence "Boxplots of these metabolites were generated with ggplot2" should be placed after these analytical steps. Furthermore, the phrase "with basic R scripts" in line 127 is unnecessary and non-standard; it should be revised to simply state that analyses were performed using R.

4. Line 177 “With 3 metabolites being higher in back fat at 180d and 360d, five metabolites increased at various stages, and 6 metabolites decreased from 160d to 360d (Fig 3)”. However, the specific identities of these metabolites are not mentioned in the text. Please add the names of these metabolites in the manuscript to point out which metabolites you are mentioned. Alternatively, clearly label the metabolites in Figures 3, 4, and 5 (e.g., Fig 3 A-B or Fig 3 C-D), and refer to them accordingly in the main text to improve clarity and traceability.

5. In the figure legends of Figures 3, 4, and 5, the sample size annotation "(n=6)" should be placed at the end of the legend, rather than immediately after the title.

In conclusion

1. Line 32 and 435 “This study provides the first comprehensive metabolomic profiling of back fat in Ningxiang pigs across developmental stages”. The use of "first" should be used with caution, as you have cited several previous studies on metabolomic profiling in pigs or related contexts. Please revise this claim in abstract and conclusion to ensure it is accurate and appropriately qualified.

Reviewer #3: The authors have addressed many reviewer questions, but important methodological, reporting and interpretive problems remain that prevent the paper from being acceptable in its present form.

Abstract

Lines 23-24: “A total of 154 metabolites exhibited significant temporal variation (p<0.05).”…. Please clarify whether the count of 154 metabolites refers to raw p-values (p < 0.05) or to FDR-adjusted q-values. Because untargeted metabolomics tests thousands of features, multiple testing correction is essential.

Materials and Methods

Lines 116-118: “A pooled quality control (QC) sample was injected after every 10 samples to monitor instrument stability and reproducibility during LC-MS analysis.”….. Please report QC metrics (e.g., PCA of QC clustering, RSD cutoff and % features removed, TIC stability, and features retained) in the main text or Supplement. If no RSD filter was applied, justify. These details are needed to support data quality claims.

Lines 163-164: “P-values were adjusted using the Benjamini-Hochberg correction.”…. The Methods state that p-values were adjusted using Benjamini–Hochberg, but the Results/Abstract report significance thresholds (p<0.05) without making clear whether quoted counts reflect adjusted q-values. Please revise all counts in the Abstract and Results so they explicitly say whether they are raw p or FDR q counts.

Results

Lines 170-171 and 188-190: “Untargeted metabolomic profiling identified 3,913 metabolites in back fat tissues of Ningxiang pigs across four developmental stages (180, 240, 300, and 360 days).” And “To identify temporally regulated metabolites and assess their functional properties, we next performed differential abundance analysis and identified 154 metabolites that significantly varied across the four developmental stages.”…………… Please distinguish between detected features (3,913) and annotated metabolites. For the 154 significant ones, indicate how many are high-confidence (MSI 1–2) vs. putative (MSI 3–4).

Lines 144-145 and 246-248: “WGCNA identified 37 different modules, each assigned with a distinct color, with highly correlated metabolites grouped together.” And “Thirty-six modules were 12 constructed (Fig 6B) and further used to build up module-trait relationships along with our previous published carcass traits [29] (Fig 7).”………..The manuscript inconsistently reports 36 vs. 37 modules. Please make this consistent, clarify whether the grey module was counted, and provide WGCNA parameters, module sizes, and the module–trait correlation matrix in the supplement.

Lines 146-147: “Our previously published carcass traits data were used to build up module-trait relationships [29].”….. Please clarify if carcass traits were measured on the same animals as the metabolomics samples. If yes, provide a table (IDs, trait values, normalization). If not, justify the use of external data and discuss the limitations of module–trait correlations.

Lines 208-214: “We also found that twenty-one metabolites were synthetic metabolites, with twelve of them accumulated from 160d to 360d, five increased at various stage, four gradually decreased in the back fat from 160d to 360d (Fig 5). In addition, we identified two mycotoxins, hydrolyzed fumonisin B1 (HFB1) and paspaline in the back fat. HFB1 was higher in the back fat at 160d and 360d, but paspaline was decreased from 240d (S1 File). The function of remaining metabolites was unknown due to lack of information in the database. Detailed information was listed in S1 File”……The Results section refers to 160d, but Abstract and Methods specify 180d as the earliest time point. Please correct these inconsistencies and ensure all text, figures, and supplements consistently use 180d, 240d, 300d, and 360d.

References

All references should be reformatted according to the journal’s reference style. Please ensure consistency in author listing, journal names, title capitalization, punctuation, and DOI formatting.

**********

7. PLOS authors have the option to publish the peer review history of their article (what does this mean?). If published, this will include your full peer review and any attached files.

If you choose “no”, your identity will remain anonymous but your review may still be made public.

**Do you want your identity to be public for this peer review?**  For information about this choice, including consent withdrawal, please see our Privacy Policy.

Reviewer #1: No

Reviewer #2: No

Reviewer #3: **Yes:** Asfa Fatima

**********

[NOTE: If reviewer comments were submitted as an attachment file, they will be attached to this email and accessible via the submission site. Please log into your account, locate the manuscript record, and check for the action link "View Attachments". If this link does not appear, there are no attachment files.]

While revising your submission, please upload your figure files to the Preflight Analysis and Conversion Engine (PACE) digital diagnostic tool, https://pacev2.apexcovantage.com/. PACE helps ensure that figures meet PLOS requirements. To use PACE, you must first register as a user. Registration is free. Then, login and navigate to the UPLOAD tab, where you will find detailed instructions on how to use the tool. If you encounter any issues or have any questions when using PACE, please email PLOS at figures@plos.org. Please note that Supporting Information files do not need this step.

Attachment Submitted filename: PONE-D-25-26633_R2 Comments.docx

# Author comment: Author response to Decision Letter 3

**DOI:** 10.1371/journal.pone.0353743.r006

11 Nov 2025

We sincerely thank the reviewers and editors for their constructive feedback and valuable suggestions. In response, we have thoroughly revised the manuscript to improve methodological transparency, data consistency, and interpretation. The QC validation section has been expanded with detailed descriptions of feature filtering, reproducibility metrics, and instrument stability results. WGCNA module information have been carefully cross-checked to ensure internal consistency across the text, figures, and supplementary files. In addition, the language, formatting, figure labeling, and supplementary materials have been comprehensively edited to enhance clarity, accuracy, and compliance with journal standards. Point-by-point responses to all reviewer comments are provided below.

Reviewer #1:

(1) The typical slaughter age for Ningxiang pigs is 8 months. However, in the study, the pigs were raised until they reached 360 days old. Has this exceeded the standard slaughter age requirement?

Response:

We thank the reviewer for this important observation. While the typical commercial slaughter age for Ningxiang pigs is around 8 months (~240 days), the goal of our study was to investigate temporal changes in metabolomic profiles and backfat development across a broader developmental period, including late-finishing stages. Raising pigs to 360 days allowed us to capture full-body maturity and late-stage metabolic changes, which are critical for understanding fat deposition and quality traits. All procedures were conducted in strict accordance with animal welfare guidelines, and the extended rearing did not compromise the health or welfare of the animals.

(2) I think that the PCA plot only indicates that there are significant differences among the four stages, but it does not represent the conclusion described by authors.

Response:

We thank the reviewer for the comment. We agree that the PCA primarily shows overall differences in metabolomic profiles among developmental stages rather than providing mechanistic conclusions. Therefore, we have revised the text to more accurately reflect the descriptive nature of PCA.

(3) The result does not seem to cover all the classification results of non-targeted metabolites.

Response:

The results do cover all the classification results of non-targeted metabolites; however, this may not have been sufficiently clear. The same concern was also raised by Reviewer 2. To address this, we have now explicitly specified the identities of these metabolites in the Results section.

(4) If there are other references for using metabolites for co-expression analysis, please add them to the methods section.

Response:

There are many studies used metabolites for co-expression analysis, we have added a reliable reference to the methods section. The sentence reads “Further details on metabolite-based co-expression analysis can be found in previous studies integrating metabolomic data for network construction [32].”

Reviewer #2:

This study presents a metabolomic analysis of back fat in Ningxiang pigs across the fattening stage (180–360 days), a critical developmental window for lipid deposition and carcass quality. The work provided a metabolic atlas of backfat dynamics during this period in an indigenous pig breed. However, several issues require revision to improve clarity, accuracy, and rigor.

In abstract

1. Abbreviations in the text should be defined at their first appearance. For example, in the abstract (line: 30), 'weighted gene co-expression network' should be followed by their abbreviations. Additionally, 'weighted gene co-expression network' is missing the word 'analysis' (should be 'weighted gene co-expression network analysis (WGCNA). Similar issues may exist elsewhere; please conduct a thorough review of the entire manuscript.

Response:

We thank the reviewer for this helpful editorial comment. We have revised the manuscript to define all abbreviations at their first appearance and to correct wording errors.

2. Line 22–23: "metabolomic profile" should be changed to the plural form "profiles" because the study involves multiple time points.

Response:

Revised as suggested. The phrase now reads “metabolomic profiles” to reflect multiple sampling time points.

3. Line 24, the less-than sign (<) in (p<0.05) should be formatted with spaces around the operator, i.e., "(p < 0.05)", as shown on line 30. Please conduct a thorough check throughout the entire manuscript.

Response:

All instances of p-values have been checked and reformatted to include spaces, e.g., (p < 0.05), consistently throughout the manuscript.

In the Keywords, please change Sus scrofa domesticus to Ningxiang pigs

Response:

The keyword “Sus scrofa domesticus” has been replaced with “Ningxiang pigs.”

In introduction

1. In line 49: The thickness of "backfat" is a critical indicator in pig breeding and meat production". The term "backfat" appears in two different forms throughout the manuscript: "backfat" and "back fat". Please standardize and use "backfat" consistently throughout the entire text.

Response:

All instances have been standardized to “backfat.”

Line 59: Ningxiang pigs, like many Chinese indigenous pig breeds, reach sexual maturity relatively early (120 day(d)), but full body maturity occurs later (300-360d). Please delete day ().

Response:

Revised as suggested. The sentence now reads:

“Ningxiang pigs, like many Chinese indigenous pig breeds, reach sexual maturity relatively early (120 d), but full body maturity occurs later (300–360 d).”

Line 77-78: The pigs were provided with free access to clean water and a nutritionally balanced diet throughout the study. Please change "provided with" to "had ad libitum access to".

Response:

The sentence has been revised to:

“The pigs had ad libitum access to clean water and a nutritionally balanced diet throughout the study.”

Lines 85-89: The manuscript should include a clear description of the slaughter method used. Specifically, please clarify whether electrical stunning or intravenous injection (e.g., pentobarbital) was employed for euthanasia.

Response:

We appreciate the reviewer’s attention to animal welfare details. The requested information is already included in the manuscript. The relevant sentence reads:

“The pigs were humanely euthanized using electrical stunning followed by slaughter.”

In Materials and Methods

Line 86It is recommended to provide the general composition of the diet used in the study in a supplementary table.

Response:

We thank the reviewer for this valuable suggestion. We have now added a supplementary table (Table S1) providing the ingredient composition and calculated nutrient contents of the diet used in the method.

1. Line 132: The package "ggplot" should be corrected to "ggplot2", as "ggplot" is outdated and the current standard is "ggplot2". Additionally, please provide version numbers for all software and R packages used, such as ggplot2 and WGCNA, to ensure reproducibility. In addition, the statement "Detailed ANOVA and Tukey’s HSD test results were listed in the S1 File" could be improved. It is recommended to format the supplementary materials as clearly labeled tables (e.g., Table S1, Table S2, Table S3) to enhance readability and facilitate reference.

Response:

Revised as suggested. The package name has been corrected to “ggplot2”, and version numbers for all software and R packages (e.g., R v 4.4.2, ggplot2 v3.5.2, WGCNA v1.73) have been added to ensure reproducibility.

Additionally, the supplementary materials have been reformatted into clearly labeled supplementary tables (Table S1..etc) for improved clarity and reference. However, it is impractical to reformat and present some results into tables due to the large number of data included. These results are still presented as supplementary files (excel files).

2. In line 150: Please ensure consistent use of lowercase "p" for p value throughout the manuscript (e.g., p < 0.05).

Response:

All instances of statistical significance notation have been checked and standardized to use lowercase “p” consistently throughout the manuscript (e.g., p < 0.05).

In Results

1. In line 171: Untargeted metabolomic profiling identified 3,913 metabolites in back fat tissues of 171 Ningxiang pigs across four developmental stages (180, 240, 300, and 360 days). Please provide the total number of metabolites identified at each developmental stage, and indicate in the text which supplementary table (e.g., Table S1, S2, etc.) contains the detailed metabolite data.

Response:

In our study, untargeted LC–MS/MS analysis was performed under identical chromatographic and mass spectrometric conditions for all samples. Therefore, metabolite detection was based on a unified peak alignment and identification process across all developmental stages, resulting in a single set of 3,913 metabolites detected in the overall dataset. As such, the total number of metabolites identified does not differ among the four stages (180, 240, 300, and 360 days). However, the relative intensities of many metabolites varied significantly among stages, reflecting biological differences in metabolic activity during fat deposition.

2. Please include the percentage of variance explained by each principal component (e.g., PC1 and PC2) on the axes of the PCA plot in Fig 1

Response:

The percentage of variance explained by each principal component has now been added to the axes of the PCA plot in Figure 1 (PC1: 38.77%, PC2: 17.04%, PC3:10.37%).

3. There is an incorrect sequence in the statistical description in Lines 126–131. One-way ANOVA should first be used to test whether there are significant differences among all groups (overall significance). This is followed by P-value adjustment (e.g., Benjamini-Hochberg correction). Only for metabolites showing significant results in the ANOVA (after correction) should post-hoc pairwise comparisons (e.g., Tukey’s HSD test) be performed to identify which specific group pairs differ significantly. Additionally, the sentence "Boxplots of these metabolites were generated with ggplot2" should be placed after these analytical steps. Furthermore, the phrase "with basic R scripts" in line 127 is unnecessary and non-standard; it should be revised to simply state that analyses were performed using R.

Response:

The section has been revised to clearly describe the correct sequence of analyses: one-way ANOVA for overall significance, followed by Benjamini–Hochberg adjustment, and then Tukey’s HSD test for post-hoc comparisons. The redundant phrase “with basic R scripts” was removed, and the description of boxplot generation has been placed appropriately after the statistical steps.

4. Line 177 “With 3 metabolites being higher in back fat at 180d and 360d, five metabolites increased at various stages, and 6 metabolites decreased from 160d to 360d (Fig 3)”. However, the specific identities of these metabolites are not mentioned in the text. Please add the names of these metabolites in the manuscript to point out which metabolites you are mentioned. Alternatively, clearly label the metabolites in Figures 3, 4, and 5 (e.g., Fig 3 A-B or Fig 3 C-D), and refer to them accordingly in the main text to improve clarity and traceability.

Response:

We appreciate the reviewer’s suggestion to improve clarity. The manuscript and corresponding figures have been updated to specify the identities of the metabolites mentioned in Line 177. The metabolite names have been already labelled along with the bar chart in Fig 3-5.

5. In the figure legends of Figures 3, 4, and 5, the sample size annotation "(n=6)" should be placed at the end of the legend, rather than immediately after the title.

Response:

The sample size annotation “(n = 6)” has been moved to the end of the figure legends for Figures 3, 4, and 5 to improve consistency and readability.

In conclusion

1. Line 32 and 435 “This study provides the first comprehensive metabolomic profiling of back fat in Ningxiang pigs across developmental stages”. The use of "first" should be used with caution, as you have cited several previous studies on metabolomic profiling in pigs or related contexts. Please revise this claim in abstract and conclusion to ensure it is accurate and appropriately qualified.

Response:

The phrasing has been revised in both the Abstract and Conclusion to avoid overstatement.

Reviewer #3:

The authors have addressed many reviewer questions, but important methodological, reporting and interpretive problems remain that prevent the paper from being acceptable in its present form.

Abstract

Lines 23-24: “A total of 154 metabolites exhibited significant temporal variation (p<0.05).”…. Please clarify whether the count of 154 metabolites refers to raw p-values (p < 0.05) or to FDR-adjusted q-values. Because untargeted metabolomics tests thousands of features, multiple testing correction is essential.

Response:

We thank the reviewer for the comment. We used FDR-adjusted q-values (Benjamini–Hochberg correction) to identify significantly altered metabolites. To avoid confusion, all p < 0.05 have been changed to q < 0.05.

Materials and Methods

Lines 116-118: “A pooled quality control (QC) sample was injected after every 10 samples to monitor instrument stability and reproducibility during LC-MS analysis.”….. Please report QC metrics (e.g., PCA of QC clustering, RSD cutoff and % features removed, TIC stability, and features retained) in the main text or Supplement. If no RSD filter was applied, justify. These details are needed to support data quality claims.

Response:

We appreciate the reviewer’s insightful suggestion. Pooled QC samples were injected after every ten study samples. QC assessment on raw intensities showed that 3 826 of 3 913 features (97.8 %) passed the RSD cutoff of 30 %, indicating high reproducibility. RSD values ranged from 1.0 % to 105.1 %, with a median of 9.8 % and mean of 12.1 %. Principal component analysis (PCA) revealed tight clustering of QC injections. Total ion current (TIC) was calculated for all QC injections to monitor overall signal stability. TIC values were highly consistent, with a mean of 9.02 × 10¹⁰ and a standard deviation of 1.93 × 10⁹, resulting in a coefficient of variation (CV) of 2.14 %. The TIC plot showed no systematic drift across injections, confirming excellent instrument performance and reproducibility throughout the analytical run.

No RSD-based filtering was applied because QC evaluation showed that the majority of features were highly reproducible. Median RSD across QC injections was 9.8 %, 75 % of features had RSD < 15 %, and TIC CV was 2.14 %, indicating excellent instrument stability. Moreover, retaining all detected features minimizes potential bias and preserves low-abundance but biologically relevant metabolites that might otherwise be excluded by strict RSD thresholds. Therefore, all 3,913 detected features were retained for downstream analysis.

Lines 163-164: “P-values were adjusted using the Benjamini-Hochberg correction.”…. The Methods state that p-values were adjusted using Benjamini–Hochberg, but the Results/Abstract report significance thresholds (p<0.05) without making clear whether quoted counts reflect adjusted q-values. Please revise all counts in the Abstract and Results so they explicitly say whether they are raw p or FDR q counts.

Response:

We thank the reviewer for the comment. To ensure clarity, all counts of significant metabolites in the Abstract and Results have been revised to explicitly indicate that they reflect q-values rather than raw p-values.

Results

Lines 170-171 and 188-190: “Untargeted metabolomic profiling identified 3,913 metabolites in back fat tissues of Ningxiang pigs across four developmental stages (180, 240, 300, and 360 days).” And “To identify temporally regulated metabolites and assess their functional properties, we next performed differential abundance analysis and identified 154 metabolites that significantly varied across the four developmental stages.”…………… Please distinguish between detected features (3,913) and annotated metabolites. For the 154 significant ones, indicate how many are high-confidence (MSI 1–2) vs. putative (MSI 3–4).

Response:

To clarify, the 3,913 features reported represent all detected metabolic features from untarget

Attachment Submitted filename: Response to reviewers Nov 12.docx

# Peer review: Decision Letter 3

**DOI:** 10.1371/journal.pone.0353743.r007

**Authors:** Aziz ur Rahman Muhammad, Aziz ur Rahman Muhammad, Aziz ur Rahman Muhammad, Aziz ur Rahman Muhammad

30 Mar 2026

<div>PONE-D-25-26633R3

Metabolomic profiling of backfat in Ningxiang pigsreveals lipid dynamics and carcass trait associations during the fattening stage

PLOS One

Dear Dr. Chen,

Thank you for submitting your manuscript to PLOS ONE. After careful consideration, we feel that it has merit but does not fully meet PLOS ONE’s publication criteria as it currently stands. Therefore, we invite you to submit a revised version of the manuscript that addresses the points raised during the review process.

Dear Authors, most of the comments are primarily related to grammar and language quality. Therefore, I would like to request that the authors carefully revise the manuscript to address these issues before it proceeds to formal acceptance.

Please submit your revised manuscript by May 14 2026 11:59PM. If you will need more time than this to complete your revisions, please reply to this message or contact the journal office at plosone@plos.org. When you're ready to submit your revision, log on to https://www.editorialmanager.com/pone/ and select the 'Submissions Needing Revision' folder to locate your manuscript file.

Please include the following items when submitting your revised manuscript:

- A letter that responds to each point raised by the academic editor and reviewer(s). You should upload this letter as a separate file labeled 'Response to Reviewers'.
- A marked-up copy of your manuscript that highlights changes made to the original version. You should upload this as a separate file labeled 'Revised Manuscript with Track Changes'.
- An unmarked version of your revised paper without tracked changes. You should upload this as a separate file labeled 'Manuscript'.

If you would like to make changes to your financial disclosure, please include your updated statement in your cover letter. Guidelines for resubmitting your figure files are available below the reviewer comments at the end of this letter.

If applicable, we recommend that you deposit your laboratory protocols in protocols.io to enhance the reproducibility of your results. Protocols.io assigns your protocol its own identifier (DOI) so that it can be cited independently in the future. For instructions see: https://journals.plos.org/plosone/s/submission-guidelines#loc-laboratory-protocols. Additionally, PLOS ONE offers an option for publishing peer-reviewed Lab Protocol articles, which describe protocols hosted on protocols.io. Read more information on sharing protocols at https://plos.org/protocols?utm_medium=editorial-email&utm_source=authorletters&utm_campaign=protocols.

As the corresponding author, your ORCID iD is verified in the submission system and will appear in the published article. PLOS supports the use of ORCID, and we encourage all coauthors to register for an ORCID iD and use it as well. Please encourage your coauthors to verify their ORCID iD within the submission system before final acceptance, as unverified ORCID iDs will not appear in the published article. *Only*  the individual author can complete the verification step; PLOS staff *cannot*  verify ORCID iDs on behalf of authors.

We look forward to receiving your revised manuscript.

Kind regards,

Aziz ur Rahman Muhammad

Academic Editor

PLOS One

Journal Requirements:

If the reviewer comments include a recommendation to cite specific previously published works, please review and evaluate these publications to determine whether they are relevant and should be cited. There is no requirement to cite these works unless the editor has indicated otherwise.

Please review your reference list to ensure that it is complete and correct. If you have cited papers that have been retracted, please include the rationale for doing so in the manuscript text, or remove these references and replace them with relevant current references. Any changes to the reference list should be mentioned in the rebuttal letter that accompanies your revised manuscript. If you need to cite a retracted article, indicate the article’s retracted status in the References list and also include a citation and full reference for the retraction notice.

Additional Editor Comments:

Dear Editor,

The manuscript was previously evaluated by the original reviewer, who has now reviewed the revised version and recommended minor revisions, primarily related to grammar and language quality.

Therefore, I would like to request that the authors carefully revise the manuscript to address these issues before it proceeds to formal acceptance.

[Note: HTML markup is below. Please do not edit.]

Reviewers' comments:

Reviewer's Responses to Questions

**Comments to the Author**

1. If the authors have adequately addressed your comments raised in a previous round of review and you feel that this manuscript is now acceptable for publication, you may indicate that here to bypass the “Comments to the Author” section, enter your conflict of interest statement in the “Confidential to Editor” section, and submit your "Accept" recommendation.

Reviewer #1: All comments have been addressed

Reviewer #2: All comments have been addressed

Reviewer #3: All comments have been addressed

**********

2. Is the manuscript technically sound, and do the data support the conclusions?

The manuscript must describe a technically sound piece of scientific research with data that supports the conclusions. Experiments must have been conducted rigorously, with appropriate controls, replication, and sample sizes. The conclusions must be drawn appropriately based on the data presented.

Reviewer #1: Yes

Reviewer #2: Yes

Reviewer #3: Yes

**********

3. Has the statistical analysis been performed appropriately and rigorously?

Reviewer #1: Yes

Reviewer #2: Yes

Reviewer #3: Yes

**********

4. Have the authors made all data underlying the findings in their manuscript fully available?

The PLOS Data policy requires authors to make all data underlying the findings described in their manuscript fully available without restriction, with rare exception (please refer to the Data Availability Statement in the manuscript PDF file). The data should be provided as part of the manuscript or its supporting information, or deposited to a public repository. For example, in addition to summary statistics, the data points behind means, medians and variance measures should be available. If there are restrictions on publicly sharing data—e.g. participant privacy or use of data from a third party—those must be specified.

Reviewer #1: Yes

Reviewer #2: Yes

Reviewer #3: Yes

**********

5. Is the manuscript presented in an intelligible fashion and written in standard English?

PLOS ONE does not copyedit accepted manuscripts, so the language in submitted articles must be clear, correct, and unambiguous. Any typographical or grammatical errors should be corrected at revision, so please note any specific errors here.

Reviewer #1: Yes

Reviewer #2: Yes

Reviewer #3: Yes

**********

6. Review Comments to the Author

Please use the space provided to explain your answers to the questions above. You may also include additional comments for the author, including concerns about dual publication, research ethics, or publication ethics. (Please upload your review as an attachment if it exceeds 20,000 characters)

Reviewer #1: #1 Should the term "back fat" in the full text be corrected to "backfat"?

#2 Carcass traits do not include indicators such as organ weight and length.

#3 Is it necessary to supplement the genetic background of 24 pigs?

#4 Line 137: The DOI address is displayed as "Not found". Please verify and make necessary modifications.

#5 It is recommended to place data such as carcass traits in the appendix.

Reviewer #2: I have reviewed the revised version of this manuscript. The authors have conscientiously addressed the previous comments and made substantial improvements to the paper. The revised manuscript shows significant enhancements in experimental validation and language quality. Therefore, I recommend this manuscript for acceptance.

Reviewer #3: The overall quality of the manuscript is good and the raised issues have been addressed properly; however, several minor language and wording issues should be corrected to improve readability. The specific suggestions are listed below.

Line 110: “Metabolites eluted from the column was detected with a high-resolution tandem mass spectrometer” replace with “Metabolites eluted from the column were detected with a high-resolution tandem mass spectrometer”

Line 214: Replace “methanandamide phosphate was deceased at 240d” with “methanandamide phosphate was decreased at 240d”

Line 221: Rephrase the sentence “Twelve were bioactive metabolites, including signaling alkaloids and antioxidants, were also temporally regulated” to “Twelve bioactive metabolites, including signaling alkaloids and antioxidants, were also temporally regulated.”

Line 266: “a cut-off of R² = 0.9” rewrite as “A cut-off of R² = 0.9”

Line 441: “While direct evidence linking pseudouridine to energy homeostasis is limited, but its synthesize enzyme, pseudouridine synthase,”…. It is “its synthesizing enzyme”.

**********

7. PLOS authors have the option to publish the peer review history of their article (what does this mean?). If published, this will include your full peer review and any attached files.

If you choose “no”, your identity will remain anonymous but your review may still be made public.

**Do you want your identity to be public for this peer review?**  For information about this choice, including consent withdrawal, please see our Privacy Policy.

Reviewer #1: No

Reviewer #2: No

Reviewer #3: **Yes:** Asfa Fatima

**********

[NOTE: If reviewer comments were submitted as an attachment file, they will be attached to this email and accessible via the submission site. Please log into your account, locate the manuscript record, and check for the action link "View Attachments". If this link does not appear, there are no attachment files.]

To ensure your figures meet our technical requirements, please review our figure guidelines: https://journals.plos.org/plosone/s/figures

You may also use PLOS’s free figure tool, NAAS, to help you prepare publication quality figures: https://journals.plos.org/plosone/s/figures#loc-tools-for-figure-preparation.

NAAS will assess whether your figures meet our technical requirements by comparing each figure against our figure specifications.

Attachment Submitted filename: PONE-D-25-26633_R3 comments.docx

# Author comment: Author response to Decision Letter 4

**DOI:** 10.1371/journal.pone.0353743.r008

1 Apr 2026

Reviewer #1:

#1 Should the term "back fat" in the full text be corrected to "backfat"?

We thank the reviewer for this suggestion. We have revised the manuscript by correcting all instances of 'back fat' to 'backfat', including those in the figures.

#2 Carcass traits do not include indicators such as organ weight and length.

These organ weight and length data (heart weight, lung weight, intestine length etc..) were already included in our experiment, as shown in Figure 7 and File S4.

#3 Is it necessary to supplement the genetic background of 24 pigs?

We have supplemented the genetic background information in the revised manuscript. It now reads “The 24 Ningxiang pigs at four developmental stages (180, 240, 300, and 360 days) were provided by Hunan Liushahe Ecological Animal Husbandry Co., Ltd., and all originated from the same closed breeding population to ensure genetic consistency.”

#4 Line 137: The DOI address is displayed as "Not found". Please verify and make necessary modifications.

We apologize for this. We have corrected the DOI.

#5 It is recommended to place data such as carcass traits in the appendix.

All raw carcass trait data were already included in our File S4.

Reviewer #2: I have reviewed the revised version of this manuscript. The authors have conscientiously addressed the previous comments and made substantial improvements to the paper. The revised manuscript shows significant enhancements in experimental validation and language quality. Therefore, I recommend this manuscript for acceptance.

We sincerely thank the reviewer for the positive feedback and for acknowledging the improvements made in the revised manuscript. We appreciate the reviewer’s time and expertise in evaluating our work.

Reviewer #3: The overall quality of the manuscript is good and the raised issues have been addressed properly; however, several minor language and wording issues should be corrected to improve readability. The specific suggestions are listed below.

We thank the reviewer for the positive assessment and for providing specific language and wording suggestions. We have carefully incorporated all of these corrections to enhance the manuscript's readability. Our point-by-point responses are as follows:

Line 110: “Metabolites eluted from the column was detected with a high-resolution tandem mass spectrometer” replace with “Metabolites eluted from the column were detected with a high-resolution tandem mass spectrometer”

We have corrected this grammatical error as suggested. The sentence now reads: “Metabolites eluted from the column were detected with a high-resolution tandem mass spectrometer.”

Line 214: Replace “methanandamide phosphate was deceased at 240d” with “methanandamide phosphate was decreased at 240d”

We thank the reviewer for catching this typo. We have replaced "deceased" with "decreased" in the revised manuscript.

Line 221: Rephrase the sentence “Twelve were bioactive metabolites, including signaling alkaloids and antioxidants, were also temporally regulated” to “Twelve bioactive metabolites, including signaling alkaloids and antioxidants, were also temporally regulated.”

We have rephrased the sentence as recommended. The corrected sentence now reads: “Twelve bioactive metabolites, including signaling alkaloids and antioxidants, were also temporally regulated.”

Line 266: “a cut-off of R² = 0.9” rewrite as “A cut-off of R² = 0.9”

We have revised this sentence to begin with a capital letter, as suggested.

Line 441: “While direct evidence linking pseudouridine to energy homeostasis is limited, but its synthesize enzyme, pseudouridine synthase,”…. It is “its synthesizing enzyme”.

We have corrected this sentence. The revised text now reads: “While direct evidence linking pseudouridine to energy homeostasis is limited, its synthesizing enzyme, pseudouridine synthase, …”

Attachment Submitted filename: Response to reviewers Mar 31.docx

# Editor report: Decision Letter 4

**DOI:** 10.1371/journal.pone.0353743.r009

**Authors:** Aziz ur Rahman Muhammad, Aziz ur Rahman Muhammad, Aziz ur Rahman Muhammad, Aziz ur Rahman Muhammad, Aziz ur Rahman Muhammad

13 Apr 2026

PONE-D-25-26633R4

Metabolomic profiling of backfat in Ningxiang pigs reveals lipid dynamics and carcass trait associations during the fattening stage

PLOS One

Dear Dr. Chen,

Thank you for submitting your manuscript to PLOS ONE. After careful consideration, we feel that it has merit but does not fully meet PLOS ONE’s publication criteria as it currently stands. Therefore, we invite you to submit a revised version of the manuscript that addresses the points raised during the review process by editor.

Please submit your revised manuscript by May 28 2026 11:59PM. If you will need more time than this to complete your revisions, please reply to this message or contact the journal office at plosone@plos.org. When you're ready to submit your revision, log on to https://www.editorialmanager.com/pone/ and select the 'Submissions Needing Revision' folder to locate your manuscript file.

Please include the following items when submitting your revised manuscript:

- A letter that responds to each point raised by the academic editor and reviewer(s). You should upload this letter as a separate file labeled 'Response to Reviewers'.
- A marked-up copy of your manuscript that highlights changes made to the original version. You should upload this as a separate file labeled 'Revised Manuscript with Track Changes'.
- An unmarked version of your revised paper without tracked changes. You should upload this as a separate file labeled 'Manuscript'.

If you would like to make changes to your financial disclosure, please include your updated statement in your cover letter. Guidelines for resubmitting your figure files are available below the reviewer comments at the end of this letter.

If applicable, we recommend that you deposit your laboratory protocols in protocols.io to enhance the reproducibility of your results. Protocols.io assigns your protocol its own identifier (DOI) so that it can be cited independently in the future. For instructions see: https://journals.plos.org/plosone/s/submission-guidelines#loc-laboratory-protocols. Additionally, PLOS ONE offers an option for publishing peer-reviewed Lab Protocol articles, which describe protocols hosted on protocols.io. Read more information on sharing protocols at https://plos.org/protocols?utm_medium=editorial-email&utm_source=authorletters&utm_campaign=protocols.

As the corresponding author, your ORCID iD is verified in the submission system and will appear in the published article. PLOS supports the use of ORCID, and we encourage all coauthors to register for an ORCID iD and use it as well. Please encourage your coauthors to verify their ORCID iD within the submission system before final acceptance, as unverified ORCID iDs will not appear in the published article. *Only*  the individual author can complete the verification step; PLOS staff *cannot*  verify ORCID iDs on behalf of authors.

We look forward to receiving your revised manuscript.

Kind regards,

Aziz ur Rahman Muhammad

Academic Editor

PLOS One

Journal Requirements:

1. If the reviewer comments include a recommendation to cite specific previously published works, please review and evaluate these publications to determine whether they are relevant and should be cited. There is no requirement to cite these works unless the editor has indicated otherwise.

2. Please review your reference list to ensure that it is complete and correct. If you have cited papers that have been retracted, please include the rationale for doing so in the manuscript text, or remove these references and replace them with relevant current references. Any changes to the reference list should be mentioned in the rebuttal letter that accompanies your revised manuscript. If you need to cite a retracted article, indicate the article’s retracted status in the References list and also include a citation and full reference for the retraction notice.

Additional Editor Comments:

Dear Authros

Thank you for addressing the comments raised by the reviewers. I have carefully evaluated the revised manuscript and observed that certain sections, including the abstract, are not scientifically rigorous and, in some cases, reflect informal language. I kindly request that you thoroughly revise the manuscript to improve grammar, English expression, and overall sentence structure, ensuring a clear, formal, and scientific tone throughout. Additionally, please update the references by incorporating more recent and relevant literature, preferably from 2024-2025.

[Note: HTML markup is below. Please do not edit.]

[NOTE: If reviewer comments were submitted as an attachment file, they will be attached to this email and accessible via the submission site. Please log into your account, locate the manuscript record, and check for the action link "View Attachments". If this link does not appear, there are no attachment files.]

To ensure your figures meet our technical requirements, please review our figure guidelines: https://journals.plos.org/plosone/s/figures

You may also use PLOS’s free figure tool, NAAS, to help you prepare publication quality figures: https://journals.plos.org/plosone/s/figures#loc-tools-for-figure-preparation.

NAAS will assess whether your figures meet our technical requirements by comparing each figure against our figure specifications.

# Author comment: Author response to Decision Letter 5

**DOI:** 10.1371/journal.pone.0353743.r010

19 May 2026

Dear Editor,

Thank you very much for your careful evaluation of our revised manuscript and for your constructive comments. We sincerely appreciate your suggestions regarding the scientific rigor, language quality, and overall presentation of the manuscript.

In response to your comments, we have thoroughly revised the entire manuscript, including the abstract, to improve the scientific tone, grammar, and sentence structure. Informal expressions have been removed or rewritten to ensure a clearer, more formal, and academically appropriate style throughout the manuscript.

Furthermore, we have updated the reference list by incorporating several recent and relevant studies published in 2024–2025 to strengthen the scientific background and discussion of our work.

Thank you again for your valuable comments and consideration.

Attachment Submitted filename: Response to reviewers May 19.docx

# Editor report: Decision Letter 5

**DOI:** 10.1371/journal.pone.0353743.r011

**Authors:** Aziz ur Rahman Muhammad, Aziz ur Rahman Muhammad, Aziz ur Rahman Muhammad, Aziz ur Rahman Muhammad, Aziz ur Rahman Muhammad, Aziz ur Rahman Muhammad

29 Jun 2026

Metabolomic profiling of backfat in Ningxiang pigs reveals lipid dynamics and carcass trait associations during the fattening stage

PONE-D-25-26633R5

Dear Dr. Chen,

We’re pleased to inform you that your manuscript has been judged scientifically suitable for publication and will be formally accepted for publication once it meets all outstanding technical requirements.

Within one week, you’ll receive an e-mail detailing the required amendments. When these have been addressed, you’ll receive a formal acceptance letter and your manuscript will be scheduled for publication.

An invoice will be generated when your article is formally accepted. Please note, if your institution has a publishing partnership with PLOS and your article meets the relevant criteria, all or part of your publication costs will be covered. Please make sure your user information is up-to-date by logging into Editorial Manager at Editorial Manager® and clicking the ‘Update My Information' link at the top of the page. For questions related to billing, please contact billing support.

If your institution or institutions have a press office, please notify them about your upcoming paper to help maximize its impact. If they’ll be preparing press materials, please inform our press team as soon as possible -- no later than 48 hours after receiving the formal acceptance. Your manuscript will remain under strict press embargo until 2 pm Eastern Time on the date of publication. For more information, please contact onepress@plos.org.

Kind regards,

Aziz ur Rahman Muhammad

Academic Editor

PLOS One

Additional Editor Comments (optional):

Dear Authors

Thanks for revision

Reviewers' comments:

# Editor report: Acceptance letter

**DOI:** 10.1371/journal.pone.0353743.r012

**Authors:** Aziz ur Rahman Muhammad, Aziz ur Rahman Muhammad, Aziz ur Rahman Muhammad, Aziz ur Rahman Muhammad, Aziz ur Rahman Muhammad, Aziz ur Rahman Muhammad

PONE-D-25-26633R5

PLOS One

Dear Dr. Chen,

I'm pleased to inform you that your manuscript has been deemed suitable for publication in PLOS One. Congratulations! Your manuscript is now being handed over to our production team.

At this stage, our production department will prepare your paper for publication. This includes ensuring the following:

* All references, tables, and figures are properly cited

* All relevant supporting information is included in the manuscript submission,

* There are no issues that prevent the paper from being properly typeset

You will receive further instructions from the production team, including instructions on how to review your proof when it is ready. Please keep in mind that we are working through a large volume of accepted articles, so please give us a few days to review your paper and let you know the next and final steps.

Lastly, if your institution or institutions have a press office, please let them know about your upcoming paper now to help maximize its impact. If they'll be preparing press materials, please inform our press team within the next 48 hours. Your manuscript will remain under strict press embargo until 2 pm Eastern Time on the date of publication. For more information, please contact onepress@plos.org.

You will receive an invoice from PLOS for your publication fee after your manuscript has reached the completed accept phase. If you receive an email requesting payment before acceptance or for any other service, this may be a phishing scheme. Learn how to identify phishing emails and protect your accounts at https://explore.plos.org/phishing.

If we can help with anything else, please email us at customercare@plos.org.

Thank you for submitting your work to PLOS ONE and supporting open access.

Kind regards,

PLOS ONE Editorial Office Staff

on behalf of

Dr. Aziz ur Rahman Muhammad

Academic Editor

PLOS One
